# SAA1/TLR2 axis directs chemotactic migration of hepatic stellate cells responding to injury

**DOI:** 10.1016/j.isci.2021.102483

**Published:** 2021-04-29

**Authors:** Anteneh Getachew, Nasir Abbas, Kai You, Zhen Yang, Muzammal Hussain, Xinping Huang, Ziqi Cheng, Shenglin Tan, Jiawang Tao, Xiaorui Yu, Yan Chen, Fan Yang, Tingcai Pan, Yingying Xu, Guosheng Xu, Yuanqi Zhuang, FeiMa Wu, Yinxiong Li

**Affiliations:** 1 Institute of Public Health, Guangzhou Institute of Biomedicine and Health, Chinese Academy of Sciences, 190 Kaiyuan Avenue, Science Park, Guangzhou, Guangdong 510530, China; 2 University of China Academy of Sciences, Beijing 100049, China; 3 Key Laboratory of Regenerative Biology, South China Institute for Stem Cell Biology and Regenerative Medicine, Guangzhou Institutes of Biomedicine and Health, Chinese Academy of Sciences, Guangzhou 510530, China; 4 Guangdong Provincial Key Laboratory of Biocomputing, Guangzhou Institutes of Biomedicine and Health, Chinese Academy of Sciences, Guangzhou 510530, China; 5 Guangzhou Regenerative Medicine and Health Guangdong Laboratory, Guangzhou 510005, China

**Keywords:** Biochemistry, Cell engineering, Cell biology

## Abstract

Hepatic stellate cells (HSCs) are crucial for liver injury repair and cirrhosis. However, the mechanism of chemotactic recruitment of HSCs into injury loci is still largely unknown. Here, we demonstrate that serum amyloid A1 (SAA1) acts as a chemokine recruiting HSCs toward injury loci signaling via TLR2, a finding proven by gene manipulation studies in cell and mice models. The mechanistic investigations revealed that SAA1/TLR2 axis stimulates the Rac GTPases through PI3K-dependent pathways and induces phosphorylation of MLC (pSer19). Genetic deletion of TLR2 and pharmacological inhibition of PI3K diminished the phosphorylation of MLCpSer19 and migration of HSCs. In brief, SAA1 serves as a hepatic endogenous chemokine for the TLR2 receptor on HSCs, thereby initiating PI3K-dependent signaling and its effector, Rac GTPases, which consequently regulates actin filament remodeling and cell directional migration. Our findings provide novel targets for anti-fibrosis drug development.

## Highlights

• SAA1 serves as a chemokine to guide migration of HSCs toward injury locus
• TLR2 acts as a functional receptor for SAA1 in HSCs
• SAA1/TLR2 axis-mediated migration of HSCs operates through PI3K/Rac1 signaling
• SAA1/TLR2 axis provides a link for the cross talk between hepatocytes and HSCs

## Graphical abstract

**Figure:**
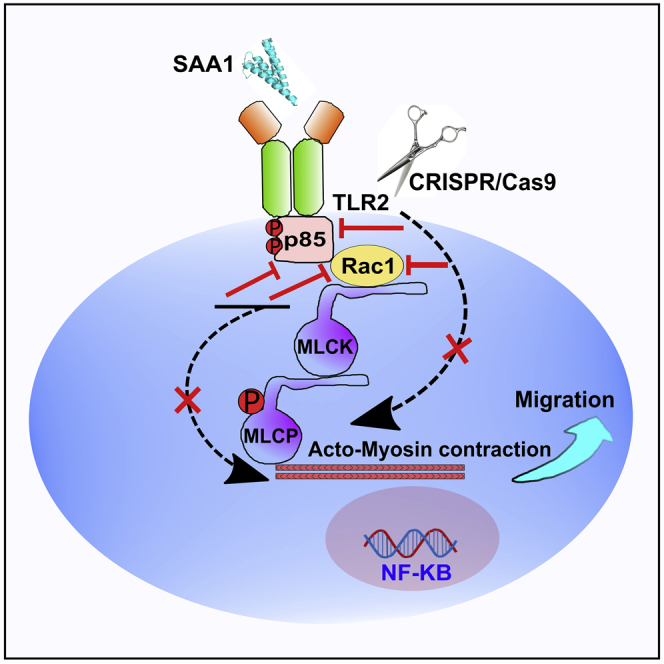

## Introduction

Hepatic fibrosis is a manifestation of wound repair in chronic liver insults resulting from overproduction of the extracellular matrix (ECM) by activated hepatic stellate cells (HSCs) (Bataller and Brenner, 2005; Mencin et al., 2009). Upon liver injury, quiescent HSCs undergo an activation process and transdifferentiate into a myofibroblast phenotype characterized by increased proliferation and migration (Border and Noble, 1994; Rojkind et al., 1983). This process is mediated by cytokines and chemokines, which are predominantly produced not only by injured hepatocytes and infiltrating immune cells but also by HSCs themselves (Geerts, 2001; Lemoinne et al., 2013). In the settings of chronic injury, HSCs continuously migrate within hepatic lobule into injury sites and deposit excessive ECM (Lemoinne et al., 2013). If this recurrent migration and deposition of ECM by HSCs cannot be shutdown, the resultant tissue scaring and neoplasia, rather than the healing process, impair the regenerative capacity and thus increase the chance for cirrhosis (Border and Noble, 1994). Therefore, due to their significant contribution in the wound healing and disease pathogenesis, HSCs have received considerable attention as a potential target to develop an effective therapy for improving liver disease outcomes (Gandhi, 2017). Despite remarkable progress in the field, however, signals mediating migration of HSCs as well as mechanisms orchestrating various facets of the fibrogenic process are still ill-defined.

Currently available literature evidence suggests that growth factors and endogenous chemokines released from dying cells may serve as the active mediators for migration of HSCs (Lemoinne et al., 2013). Of particular interest, in this regard, is serum amyloid A (SAA), one of several acute phase proteins responsible for recruiting monocytes, immature dendritic cells, phagocytes, and blood leukocytes during an inflammatory response (Badolato et al., 1995; Gouwy et al., 2015; Liang et al., 2000). SAA is abundantly produced by hepatocytes during liver inflammation or acute injury (Uhlar et al., 1994). However, the literature evidence regarding its role as a relevant promoter of hepatic inflammation and fibrogenesis remains controversial. One group reported that hepatic SAA1-induced chemokine production may exacerbate T-cell-mediated hepatitis (Ji et al., 2015), while other reports indicate that SAA1 may exhibit cytokine-like property, and it can be easily recognized by cell-to-cell communication and feedback response in inflammatory, immunogenic, and protective pathways (Baranova et al., 2005; He et al., 2003; Jijon et al., 2005). In any case, currently there has been no specific receptor reported for SAA1, although its signal transduction via multiple receptor pathways including TLR2, TLR4, FPR1, RAGE, and SR-B1 has been extensively studied (Cheng et al., 2008; Connolly et al., 2016; Liang et al., 2000; Tomita et al., 2015).

Herein, we studied the role of SAA1 in toxic CCl_4_- and cryoinjury (CI)-induced sterile acute liver injury models. Our data indicate that SAA1 exerts an essential role in recruiting HSCs into the injury sites via TLR2 signaling pathway. We initially found that SAA1 and TLR2 are simultaneously co-expressed and colocalized with HSCs in both CCl_4_ and CI models of acute liver injury. Further investigations revealed that SAA1/TLR2 axis is critical for recruitment of HSCs at the injury site, as the depletion of SAA1 or inhibiting TLR2 by antagonist significantly diminished the migration of HSCs into injury locus. We then used *in vitro* migration assays to demonstrate that SAA1 serves as a potent chemotactic signal which attracts LX-2 and rat primary HSCs via a TLR2-dependent pathway. Mechanistic investigations highlighted that migration of HSCs is firmly mediated by activation of Rac1, a member of the Rho family of small GTPases and the downstream signaling effector of PI3K. Overall, our data demonstrate that injury-induced SAA1 is engaged with its chemotactic receptor TLR2 and attracts HSCs into the injury locus.

## Results

### Gene knockdown efficiently suppressed SAA1 expression in mice models of acute liver injury

**Figure 1:**
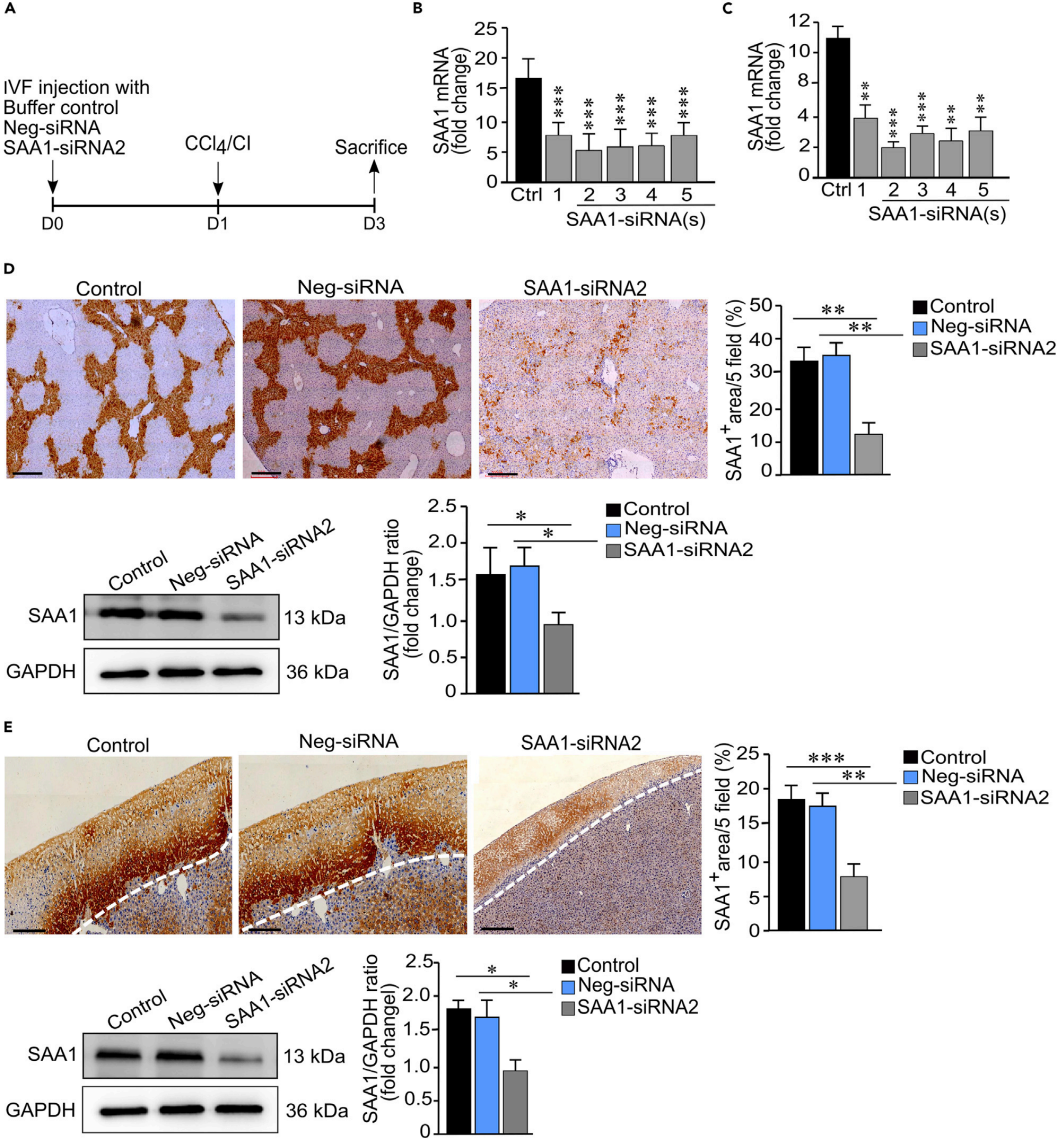
Hepatic SAA1 suppression in mice via siRNA (A) Experimental design representing suppression of SAA1 in CCl_4_ and CI injury models and timescale for sample collection. (B and C) Relative expression of liver SAA1 mRNA after 2 days of transfecting five different siRNA(s) in CCl_4_ and CI injury models, respectively. (D and E) IHC and western blot analyses of selected SAA1-siRNA2 in CCl_4_ and CI injury models, respectively. Neg-siRNA represents non-specific control. Scale bar represents 100 μm. Where applicable, data represent mean ± SEM ∗p < 0.05, ∗∗p < 0.01 and ∗∗∗p < 0.001 (n = 3).

To elucidate the specific activity of SAA1 in recruitment of HSCs responding to injury, we applied a stealth RNAi™ siRNA technology to suppress the expression of SAA1 in CCl_4_ and cryoinjury mice models. In order to achieve efficient genetic knockdown, we initially focused on identification and selection of target sequence. For this purpose, we synthesized 5 representative sequences (Table S1) and transfected them in C56BL/6 mice liver one day before CCl_4_ intraperitoneal (IP) injection, and our recently reported a localized cryoinjury model to induce acute liver injuries (Abbas et al., 2020) (Figure 1A). After 2 days of transfection, the mRNA expression of SAA1 was significantly downregulated in case of SAA1-siRNA2 in both injury models (Figures 1B and 1C). Further analyses of the selected sequence (SAA1-siRNA2), by using immunohistochemistry, quantitative RT-qPCR, and western blotting, revealed abundant hepatic expression of SAA1 in buffer and negative-control (Neg-siSAA)-treated mice which reached the peak level at 24 hr after injury; however, the level of SAA1 remained significantly declined in SAA1-siRNA2-treated mice, (Figures 1D and 1E).

### SAA1 is a chemokine attracting HSCs into the injury locus

**Figure 2:**
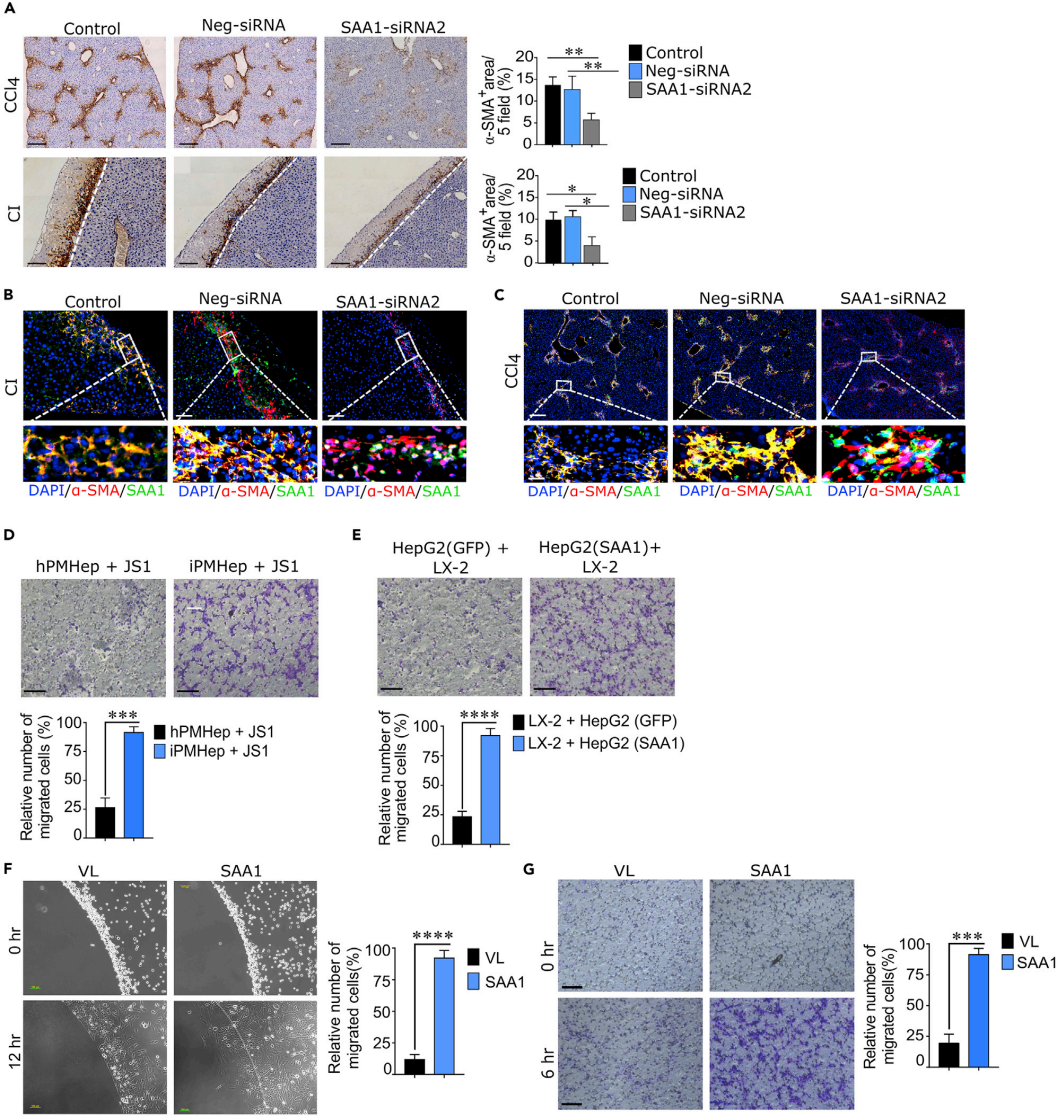
SAA1 promotes HSC recruitment *in vitro* and *in vivo* (A) Representative IHC immunostaining images showing staining of α-SMA-positive cells in SAA1-siRNA2, Neg-siRNA, and control samples obtained from CCl_4_ and CI injury models. Bar graph represents quantification of IHC images per 5 fields. (B and C) Representative confocal immunofluorescence images showing co-localization of SAA1 and HSCs in SAA1-siRNA2, Neg-siRNA, and control in CCl_4_ and CI injury models. (D and E) Transwell migration assay representing co-culture of primary mouse hepatocytes (PMHep) isolated from injured and healthy mouse with JS1 cells (D) and HepG2 cells (SAA1 overexpressing and GFP expressing) with LX-2 (E). The bar graph represents quantification of relative number of migrated cells. (F and G) Agarose spot (F) and Transwell (G) migration assays showing migration of LX-2 cells toward rhSAA1 at indicated time intervals. The bar graph represents quantification of relative number of migrated cells. (Scale bar = A, B, and C/200; G/100 μm: Inset/50 μm). Where applicable, data represent mean ± SEM ∗p < 0.05, ∗∗p < 0.01, ∗∗∗p < 0.001 and (n = 3). iPMHep represents injured primary mouse hepatocytes, hPMHep represents healthy primary mouse hepatocytes, HepG2 (SAA1) represents HepG2 cells overexpressing SAA1, and HepG2 (GFP) represents HepG2 expressing GFP.

We next assessed the expression and physiopathological relevance of SAA1, TLR2, and HSCs following hepatic injury. Both injury models resulted in abundant hepatic expression of SAA1 which reached the peak level at day 2 after the injury (Figures S1A and S1B) and then significantly declined on day 5. From IHC analysis, we found that SAA1 expression was accompanied by considerable recruitment of activated HSCs within 2 days after injury induction (Figures S1A and S1B). Furthermore, increased TLR2 expression was also evident, and it was consistent with that of SAA1 expression up to day 3 and the recruitment of HSCs (Figures S1A and S1B). Especially in case of CI-induced model, the TLR2 expression was prominent at the border edge of the wound, the area which characterized abundant expression of SAA1 and the deployment of HSCs (lower right panel in Figure S1B). This injury-induced molecular and cellular spatiotemporal appearing synchronization pattern implied a functional relation between SAA1 and TLR2 with reference to directional recruitment of HSCs to wounded area. Confocal microscopy also revealed a complete co-localization of SAA1, TLR2, and α-SMA at the injury sites (Figures S1C and S1D), a result consistent with the concept that colocalization refers to biological interactions (Manders et al., 1992). These observations prompted us to reason that SAA1, being a chemokine, probably mediates migration of HSCs following acute injury. To confirm this hypothesis, we sought to demonstrate the role of SAA1 in the recruitment of HSCs *in vivo*. As shown in Figure 2A, the recruitment of HSCs was significantly reduced (p < 0.01) in SAA1-siRNA2-treated samples as compared to the Neg-siRNA-treated and control ones. In addition, the SAA1-siRNA2 transfection also altered the co-localization of SAA1 with HSCs (Figures 2B and 2C). Thereafter, an additional *in vitro* experiment involving two separate co-culture systems demonstrated the recruitment of JS1 and LX-2 cells toward primary mouse hepatocytes—derived from injured mice liver (iPMHep)—and SAA1-overexpressing HepG2 cells, respectively (Figures 2D and 2E). These findings were consistent with our preceding hypothesis that SAA1 released from injured hepatocytes mediates recruitment of HSCs toward injury locus. This notion was further strengthened by our *in vitro* agarose spot and Transwell migration assays, in which, LX-2 cells showed migration toward rhSAA1 in dose-dependent fashion, with maximal activity observed at 10 μg/mL (Figures 2F, 2G, and S2, and Videos S1 and S2). Collectively, the above described data suggested that SAA1 is not mere a key acute phase protein but also serves as a chemokine that triggers migration of HSCs toward injury locus.

Video S1. LX-2 cells were scrambled under agarose spot containing rhSAA1, related to Figure 1

Video S2. LX-2 cells were unable to scramble under agarose spot containing PBS, related to Figure 1

### TLR2 is a receptor for SAA1 in HSCs

**Figure 3:**
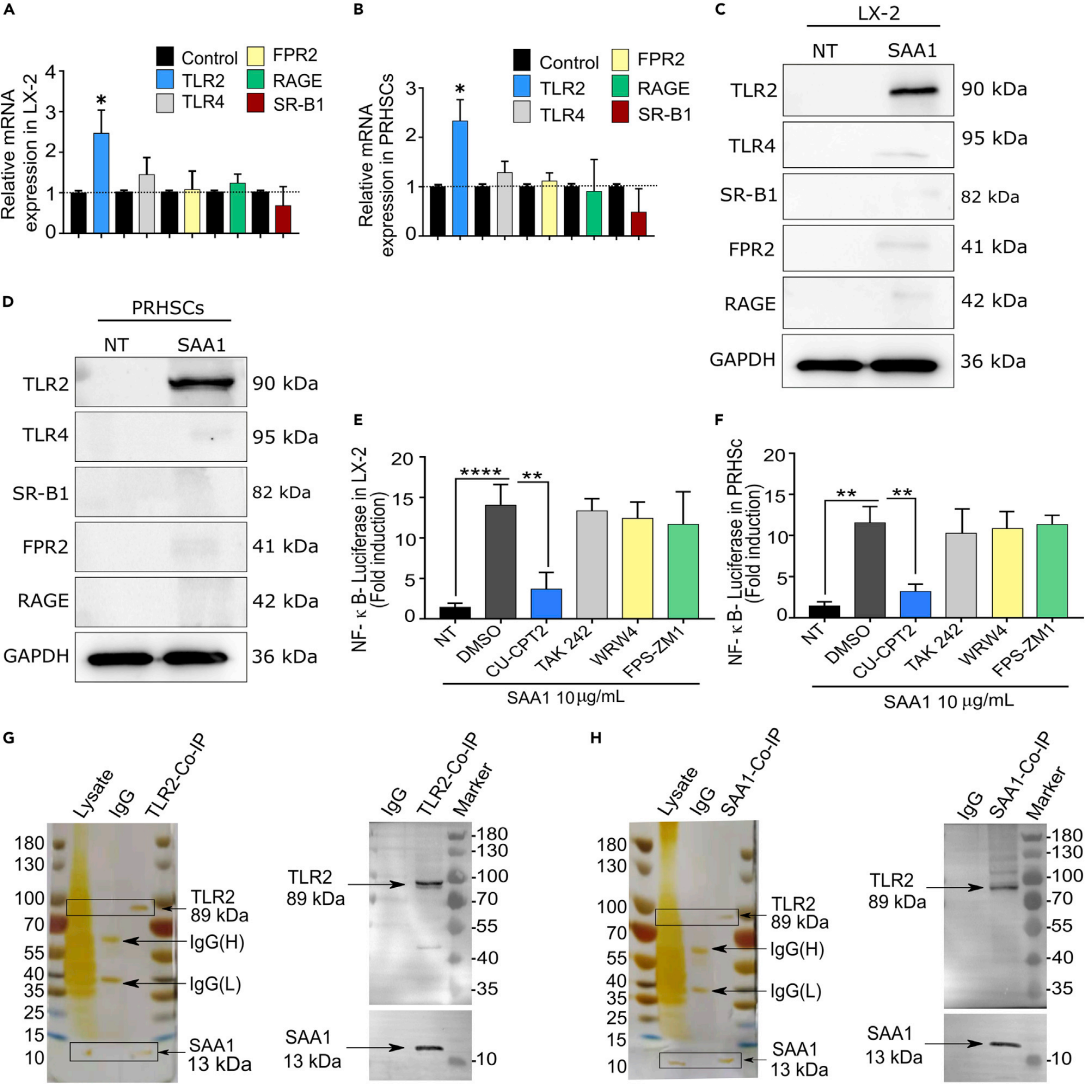
Screening of a receptor target for SAA1 in HSCs (A and B) LX-2 and PRHSCs were stimulated with rhSAA1 or rmSAA1, respectively, for 24 hr, and mRNA levels of TLR2, TLR4, FPR2, RAGE, and SR-B1 were determined by RT-qPCR. (C and D) Representative western blot analysis of receptors TLR2, TLR4, FPR2, RAGE, and SR-B1 after rhSAA1 and rmSAA1 treatment of LX-2 and PRHSCs, respectively. (E and F) LX-2 and PRHSCs were transfected with reporter plasmid for NF-κB. Twenty four hr later, the medium was changed and cells were pretreated with inhibitors of TLR2 (CU-CPT22, 1 μM), RAGE (FPS-ZM1, 0.5 μM), TLR4 (TAK 242, 5 nM), and FPR2 (WRW4, 0.25 μM) and then stimulated with rhSAA1 and rmSAA1, respectively, diluted in serum-free DMEM. Twelve hr later, luciferase activity was measured in fold induction after normalization with *Renilla* luciferase (Rluc) control. (G and H) Co-immunoprecipitation (co-IP) experiment showing the interaction between SAA1 and TLR2 determined by silver staining and western blot analysis. (For detailed information please see STAR methods section). (n = 3). Where applicable, data represent mean ± SEM ∗p < 0.05, ∗∗p < 0.01, ∗∗∗p < 0.001 and (n = 3).

Currently, there is no specific receptor reported for SAA1, albeit its functioning via multiple receptor pathways, including TLR2, TLR4, RAGE, and FPR2, has been demonstrated in different cell types (Badolato et al., 1994; Cheng et al., 2008; Liang et al., 2000). We conducted RT-qPCR and western blot analyses for these receptors in rhSAA1- and rmSAA1-treated LX-2 cells and primary rat hepatic stellate cells (PRHSCs), respectively. The results showed that the expression of TLR2 was particularly higher at both mRNA and protein levels (Figures 3A–3D). In addition, CU-CPT22, a specific inhibitor of TLR2, significantly inhibited not only the TLR2-mediated activation of NF-κB in a luciferase activity assay (Figures 3E and 3F) but also the migration of LX-2 cells (Figure S3). However, the inhibitors for other receptors failed to do so (Figures 3E, 3F, and S3). Next, to confirm whether TLR2 is a functional receptor for SAA1 in HSCs, we performed a co-immunoprecipitation assay involving pull-down of either SAA1 or TLR2 from the cellular lysates of LX-2 cells. While both proteins were able to pull down each other, there was nothing observed in case of negative (IgG) control samples (Figures 3G and 3H). This result rendered clear evidence that SAA1 and TLR2 are indeed interlinked. Overall, these findings suggested that TLR2 is a functional receptor for SAA1 in HSCs.

### SAA1/TLR2 axis mediates migration of HSCs

**Figure 4:**
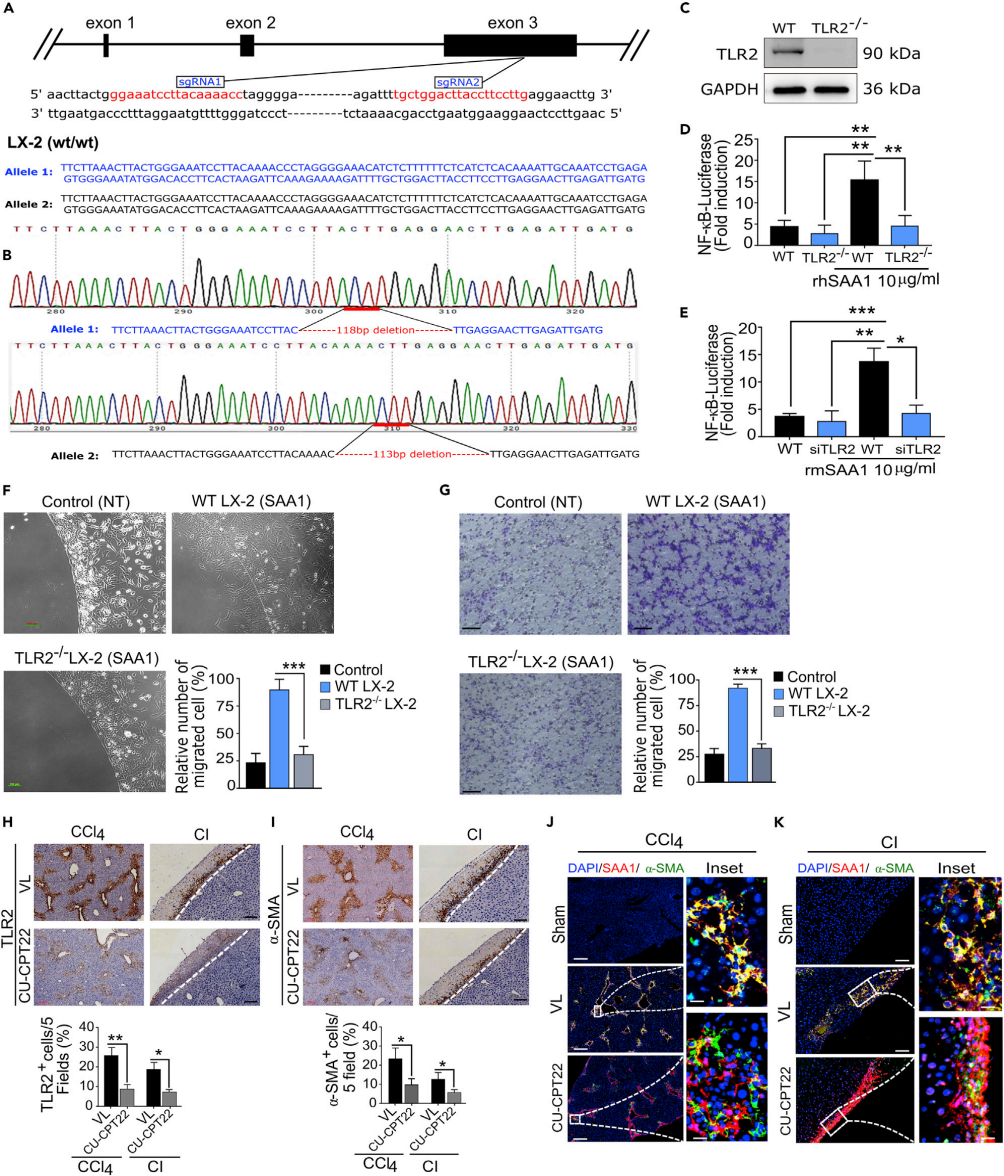
TLR2 serves as a chemotactic receptor for SAA1 and mediates migration of HSCs (A) Schematic of the TLR2 gene knockout strategy. The Cas9/sgRNA(s) target site(s) are indicated in red and were confirmed by sequencing. (B) Sequencing result of targeted region. (C) Confirmation of TLR2 gene KO at protein level determined by western blot. (D and E) WT and TLR2^−/−^ LX-2 cells (D) and WT and siPRHSCs (E) were transfected with reporter plasmid for NF-κB. Luciferase activity was measured as fold induction in comparison to untreated control (See also STAR methods section for detail information). (F and G) Representative images showing agarose spot and Transwell migration assays of SAA1-treated WT LX-2, TLR2^−/−^ LX-2 cells, and non-treated control. (H and I) Representative images for immunostaining of TLR2 (H) and α-SMA^+^ cells (I) in CU-CPT22- or VL-treated samples as shown in CCl_4_ and CI injury models. Bar graph represents quantification of IHC images per 5 field(s). (J and K) Immunofluorescence images showing co-localization of SAA1 and HSCs in CU-CPT22-treated and control (VL) samples in CCl_4_ (J) and CI injury models (K). (Scale bar represents 100 μm for CCl_4_ injury, 200 μm for CI injury, and 50 μm for inset). Data represent mean ± SEM ∗p < 0.05, ∗∗p < 0.01 and ∗∗∗p < 0.0001 (n = 3). (For detailed TLR2 knockout strategy, see STAR methods section).

Given that TLR2 may act as a functional receptor for SAA1 in HSCs, we questioned if SAA1/TLR axis may guide directional migration of HSCs toward injury locus. To answer this question, we employed genetic approaches and performed a series of cell-based *in vitro* and *in vivo* experiments. First, we generated a homozygous TLR2 knockout LX-2 cell line (TLR2^−/−^) (Figures 4A–4C, and see STAR methods section for detail information) and measured the ability of SAA1 to trigger TLR2-mediated activation of NF-κB in these cells. Interestingly, SAA1 treatment failed to induce considerable NF-κB activity in the TLR2^−/−^ LX-2 cells (Figure 4D). Similarly, siRNA-mediated silencing of TLR2 in PRHSCs (siTLR PRHSCs) also demonstrated a similar result (Figure 4E). This suggested that SAA1-induced activation of NF-κB was absolutely dependent upon TLR2. Moreover, we determined the potential of TLR2 in mediating SAA1-triggered migration of TLR2^−/−^ LX-2 cells in the *in vitro* migration assays. As shown in Figures 4F and 4G and Video S3, TLR2^−/−^ LX-2 cells were unable to migrate in response to SAA1 in both agarose spot and Transwell migration assays, respectively. We next induced *in vivo* inhibition of TLR2 by IP injection of CU-CPT22 (Figure 4H), which resulted in a significantly reduced recruitment of HSCs in CCl_4_ and CI injuries (Figure 4I); however, it did not affect the expression of SAA1 at injury sites (data not shown). Confocal microscopy further showed that SAA1 was dominantly expressed at injury sites, although the colocalization ratio between SAA1 and α-SMA was significantly altered (Figures 4J and 4K).

Video S3. TLR2^−/−^ LX-2 cells could not scramble under agarose spot containing rhSAA1, related to Figure 3

### SAA1/TLR2 axis mediates homing of engrafted HSCs toward injury locus

**Figure 5:**
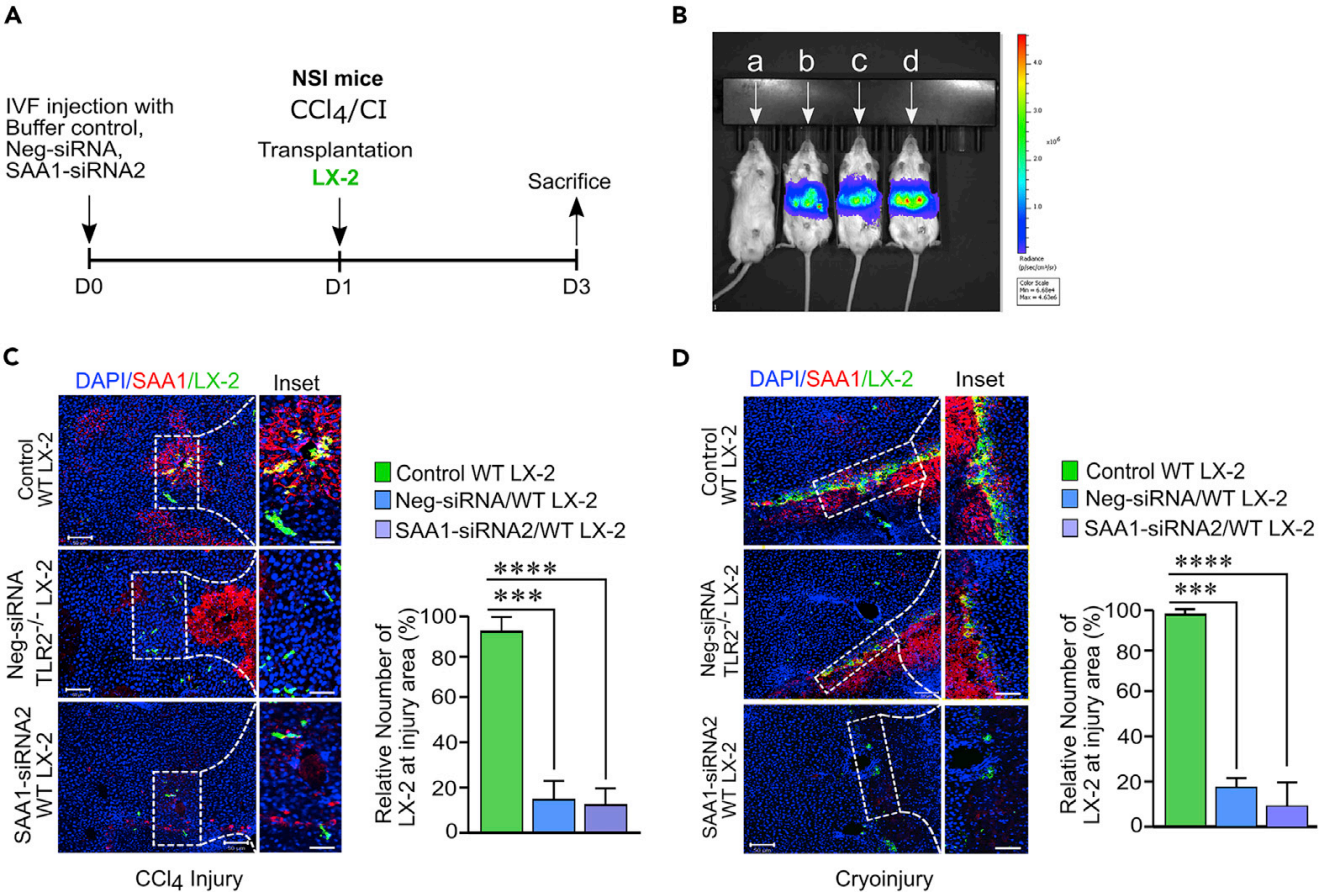
SAA1/TLR2 axis guides homing of transplanted LX-2 toward injury site(s) (A) Schematic representation of experimental design. (B) Bioluminescence imaging of transplanted cells entrapped to the liver 24 hr after transplantation in CCl_4_ injury model, (A) represents sham operated mice, (B) represents SAA1siRNA2-treated mice transplanted with WT LX-2, (C) represents Neg-siRNA-treated mice transplanted with TLR2^−/−^ LX-2, and (D) represents control mice transplanted with WT LX-2 cells. (C and D) Representative confocal immunofluorescence images showing the recruitment of transplanted WT and TLR2^−/−^LX-2 cells at injury locus after CCl_4_ and CI injuries induced in SAA1-siRNA2, Neg-siRNA, and control samples, respectively. Bar graph represents quantification of relative number of homed cells at injury site(s). (Scale bar represents 100 μM). Data represent mean ± SEM ∗p < 0.01, ∗∗p < 0.01, ∗∗∗p < 0.001 and ∗∗∗∗p < 0.0001(n = 3).

To further validate our *in vitro* and *in vivo* migration data, we urged to determine the role of SAA1 in homing of transplanted LX-2 cells at injury sites following acute liver injury. For this purpose, we generated and then intrasplenically transplanted fluorescence green fluorescent protein (GFP)-tagged WT and TLR2^−/−^ LX-2 cells into the CCl_4_- and CI-challenged livers of SAA1-depleted or WT NSI mice (Figure 5A). Tracking through live imaging system as well as the confocal microscopy revealed that the significant number of WT LX-2 cells navigated via hepatic lobule, while forming clusters and more numerous occupying surfaces at the injury site(s) (Figures 5B–5D). Strikingly, depletion of SAA1 decreased (p < 0.0001) the number of LX-2 cells entrapped in the host liver, and we observed a limited number of cells at injury site(s) (Figures 5C and 5D). Moreover, analysis of the images from confocal microscopy indicated that majority of the cells were scattered in the parenchyma of recipient livers (Figures 5C and 5D). We also observed that SAA1 could not show affinity for transplanted TLR2^−/−^ LX-2 cells in the WT mice, as only few cells were residing around the injury border while majority of them were scattered in the hepatic parenchymal region (Figures 5C and 5D). These data together suggest that SAA1/TLR2 axis mediates homing of transplanted LX-2 cells toward injury locus.

### SAA1/TLR2 axis induces Rac1-mediated actin reorganization and migration of HSCs

**Figure 6:**
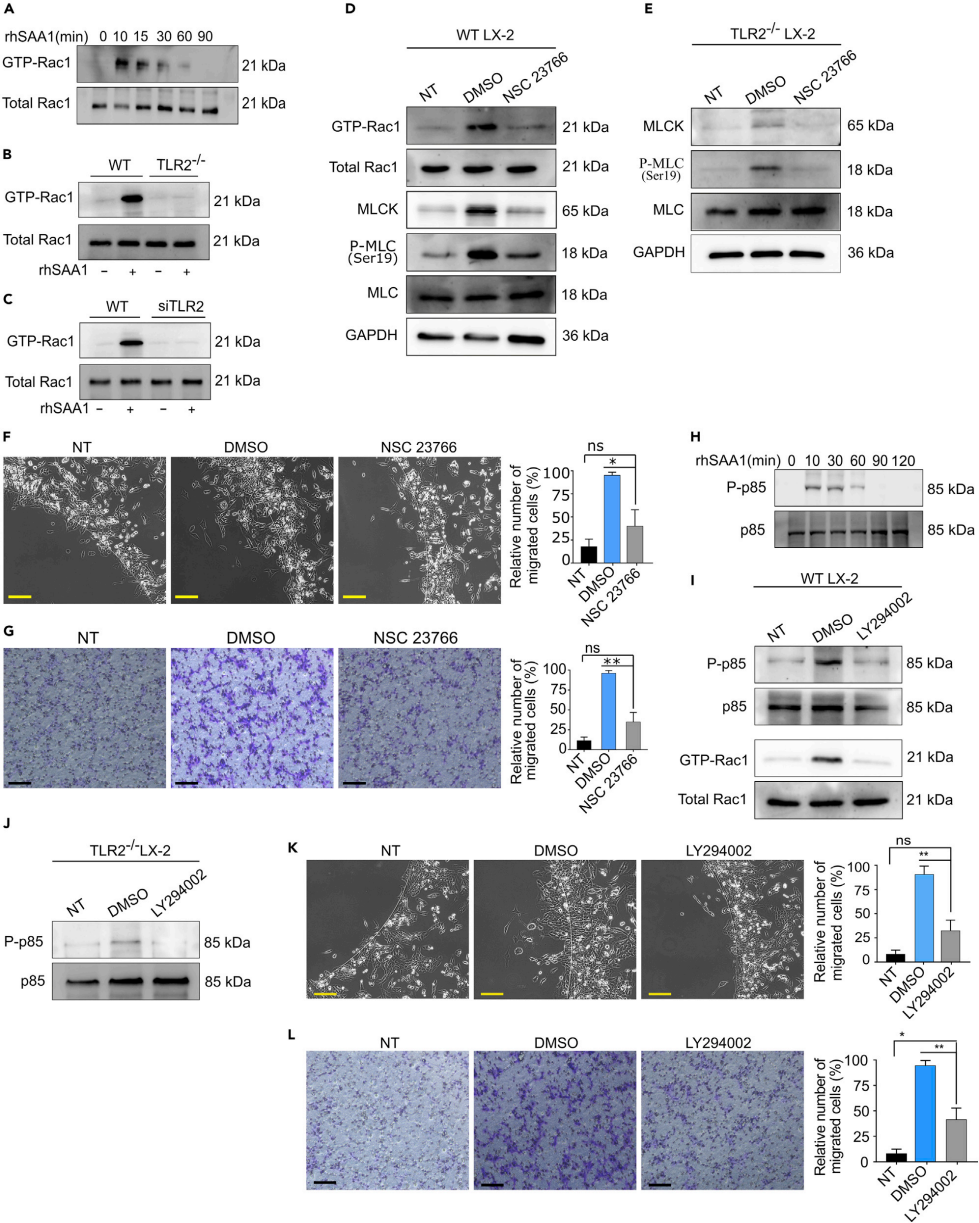
SAA1/TLR2 axis induces Rac GTPase-mediated actin reorganization and migration of HSCs (A) Time course of GTPase-Rac1 activation(s) in LX-2 cells at indicated time interval (B and C) Basal and stimulated levels of GTPase-Rac1 in WT and TLR2^−/−^ cells as determined by pull-down assays (D and E) WT and TLR2^−/−^ LX-2 cells were pretreated with NSC 23766 (50 μΜ), and activation of GTP-Rac1 was determined by pull-down assay (D), and MLCK and p-MLC at Ser19 activity was determined by western blotting (E). (F and G) Migration assays showing pretreatment of the cells with NSC 23766 (50 μΜ) attenuated their migration(s) in agarose spot (F) and Transwell (G) assays. (H) Time course of PI3K activations after treatment of LX-2 cells at indicated time points. (I and J) WT and TLR2^−/−^cells were pretreated with LY294002 (10 μΜ), and the phosphorylation of PI3K at p85 subunits was determined by western blot analysis, and the activations of Rac GTPase were determined by pull-down assay. (K and L) Migration assays showing pretreatment of the cells with LY294002 attenuated their migration(s) in agarose spot (K) and Transwell (L) assays. Photographs are representatives of (n = 6). (Scale bar represents 200 μm). Data represent mean ± SEM ∗p < 0.01 and ∗∗p < 0.001 (n = 3). Photographs are representatives of (n = 6). (Scale bar represents 200 μm). Data represent mean ± SEM ∗p < 0.01 and ∗∗p < 0.001 (n = 3).

We next sought to determine the pathways downstream of SAA1 orchestrating the migration of HSCs. Rho GTPases are a well-known regulator for multiple cellular functions including cytoskeletal reorganization, growth, proliferation, migration, and apoptosis (Lee et al., 2000). Recently, it has been reported that TLR2 coordinates with the Rho GTPases for multitude cellular activity (Arbibe et al., 2000b; Manukyan et al., 2009). We wondered whether SAA1/TLR2 axis also regulates actin cytoskeletal rearrangement in HSCs through activating this family of proteins. To test this hypothesis, we performed a pulldown assay for Rac GTPase in rhSAA1- and rmSAA1-treated LX-2 and PRHSCs, respectively. Interestingly, rhSAA1 induced time-dependent activation of Rac GTPase in LX-2 cells (Figure 6A). To further verify whether rhSAA1-induced activation of GTP-Rac1 is TLR2 dependent, we performed the pulldown assay in TLR2^−/−^ LX-2 cells and found that rhSAA1 treatment failed to induce activation of Rac1 (Figure 6B). In addition, rmSAA1 also could not induce GTP-Rac1 activation in siTLR2-treated PRHSCs (Figure 6C). Altogether, these results suggested that SAA1 induces activation of small Rac GTPase in a TLR2-dependent manner.

Previously, it has been described that Rac1 controls the myosin II activity and mediates actomyosin contraction (Han et al., 2015). We, therefore, wondered whether SAA1/TLR2-induced Rac1 activation may control the activity of myosin II. To confirm this, we checked the activation of myosin light-chain kinase (MLCK), a regulator for phosphorylation of myosin light-chain phosphatase, in the rhSAA1-treated or untreated LX-2 cells. As shown in Figure 6D, SAA1-mediated Rac1 activation (GTP-Rac1) led to increased expression of MLCK, which subsequently induced activation of MLC as indicated by enhanced expression of its phosphorylated form p-MLC^Ser19^. However, the rhSAA1 untreated cells or the rhSAA1-treated cells that were pre-treated with a pharmacological inhibitor of Rac GTPase (NSC 23766) failed to show induction of MLCK/p-MLC^Ser19^. Likewise, rhSAA1 treatment could only induce a slight expression of MLCK and p-MLC^Ser19^ in TLR2^−/−^ LX-2 cells, while the expression was completely diminished in the NSC 23766-treated cells (Figure 6E). We further evaluated the role of Rac1 in SAA1-mediated chemotaxis of LX-2 cells. Consistently, migration of LX-2 cells was significantly attenuated in the rhSAA1-untreated and pharmacologically pre-treated (but rhSAA1 treated) cells, as only a limited number of cells were observed in the cell free zone (Figures 6F and 6G). These data collectively infer that Rac1 serves an important mediator of actin reorganization and thus modulates chemotaxis of HSCs in response to a signal transduction elicited by SAA1/TLR2 axis.

PI3K is a known regulator of cell migration through cross talk with other pathways such as Rac1 GTPase (Cain and Ridley, 2009). Previously, it has been reported that PI3K coordinates actomyosin reorganization in response to PDGF-induced cell migration (Harper et al., 2007). Similarly, another study highlighted that PI3K serves upstream of Rac1 to mediate TLR2 signaling which controls the NF-kB transactivation (Arbibe et al., 2000a). Given these facts and our findings that SAA1 induces TLR2-mediated Rac 1 activation, we questioned if PI3K serves upstream of the TLR2/Rac1-derived migration of HSCs. Our western blot analysis showed that rhSAA1 treatment induced time-dependent activation of PI3K in LX-2 cells (Figure 6H). Interestingly, pretreatment of rhSAA1-stimulated LX-2 cells with pharmacological inhibitor of PI3K (LY294002) reduced the phosphorylation of p85 subunit of PI3K and thereby blocked Rac1 activation as compared to the control cells (Figure 6I). To a similar extent, rhSAA1-stimulated TLR2^−/−^ cells also demonstrated prominently less expression of phosphorylated p85 subunit (Figure 6J). This data confirmed that PI3K is upstream of Rac1 in SAA1/TLR2-derived signal transduction pathway. Next, we evaluated the effect of PI3K inhibition in SAA1-induced migration of HSCs. The results indicated that migration of LX-2 cells was compromised after incubation with LY294002 (Figures 6K and 6L). This implies that PI3K serves upstream of SAA1-induced TLR2 signal transduction that reorganizes the cell machinery, while Rac1 plays a major role downstream of PI3K.

### SAA1/TLR2 axis-mediated recruitment of HSCs increases ECM deposition at injury locus

**Figure 7:**
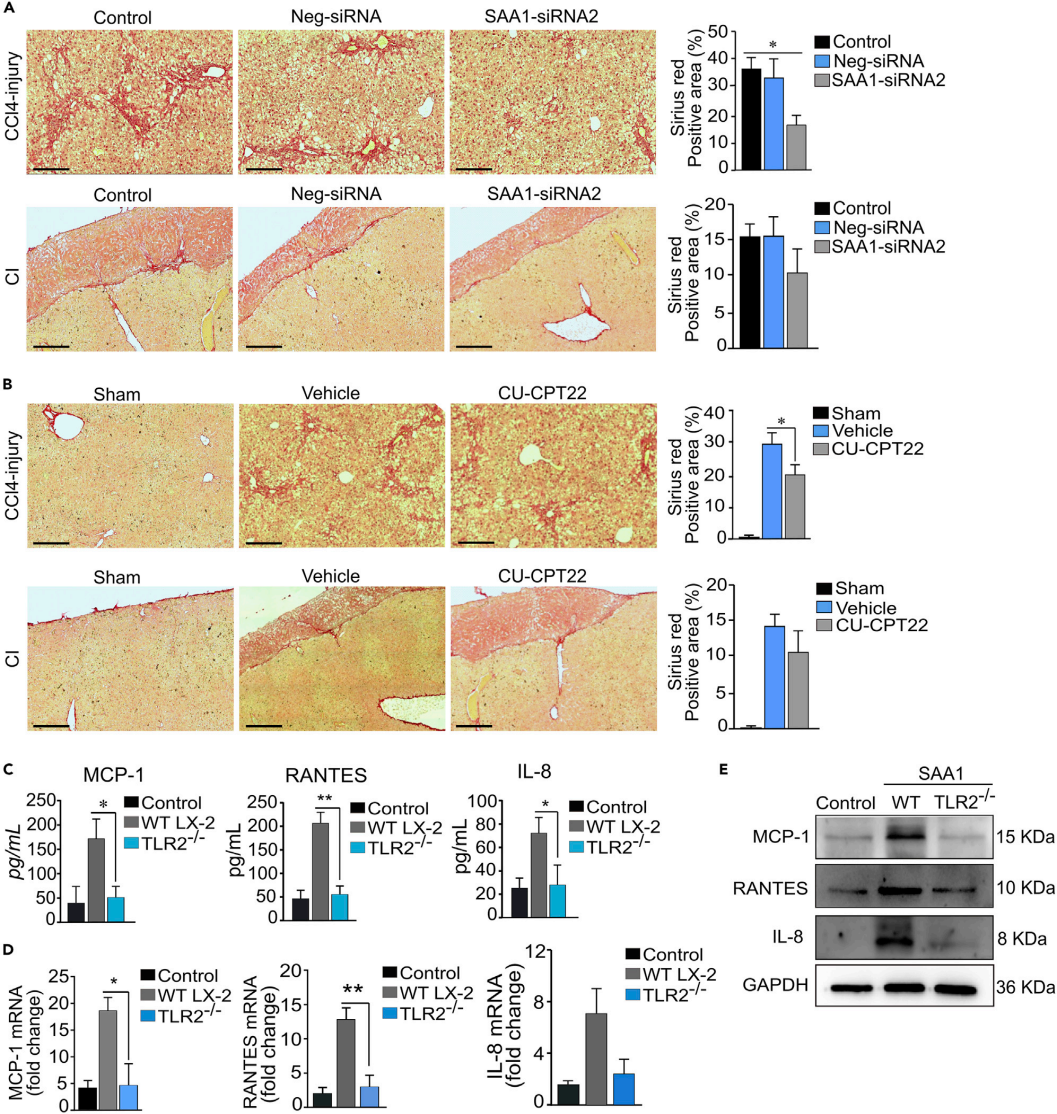
SAA1/TLR2 axis mediates increased deposition of ECM at injury sites and induces chemokines secretion in activated HSCs (A and B) Sirius red staining of ECM deposition at injury locus in CCl_4_ and Cl-induced models. (C) ELISA detection of MCP-1, IL-8, and RANTES secretion from LX-2 cells after treatment of the cells with rhSAA1 for 24 hr. (D) mRNA levels of MCP-1, IL-8, and RANTES. (E) Protein-level detection of MCP-1, IL-8, and RANTES. (Scale bar represents 100 μm). Data represent mean ± SEM ∗p < 0.05, ∗∗p < 0.01 (n = 3).

Migration of HSCs is critical for the accumulation of HSCs at the injury locus, whereby they produce ECM leading to characteristic patterns of collagen deposition during fibrogenesis (Lee and Friedman, 2011). We next sought to determine whether SAA1/TLR2 axis-mediated recruitment of HSCs could increase deposition of matrix ECM collagen (prominent ECM component of tissue fibrosis) at injury sites. For this purpose, we conducted Sirius red staining of tissue sections obtained after induction of CCl_4_ and CI injuries in SAA1- and TLR2-deficient mice liver. The analyses revealed markedly reduced ECM deposition in both SAA1- and TLR2-deficient mice liver samples, providing a reciprocal evidence for the SAA1/TLR2-mediated collagen deposition probably via recruitment of excessive activated HSCs into the injury locus (Figures 7A and 7B). We also found that rhSAA1 strongly induced the secretion of IL-8, MCP-1, and RANTES, the prominent inflammatory chemokines which are tightly associated with hepatic fibrogenesis. Interestingly, the levels of these chemokines, both in supernatant, protein, and mRNA, were significantly declined in TLR2^−/−^ and non-treated control cells, while they were significantly upregulated in rhSAA1-treated LX-2 cells (Figures 7C–7E). These results suggested that SAA1/TLR2 axis also contributes for hepatic fibrogenesis through increased deposition of ECM and induction of proinflammatory chemokines in activated HSCs.

## Discussion

SAA is the most abundantly expressed acute phase protein during liver injury. Human SAA1 and SAA2, as well as the murine SAA1, SAA2, and SAA3, are the main acute phase SAA proteins, whereas SAA4 is constitutively expressed (Uhlar et al., 1994). Currently existing literature evidence indicates that SAA1 not only induces proliferation of HSCs but also stimulates their inflammatory chemokine production (Siegmund et al., 2016). However, unlike the other chemokines such as RANTES and MCP-1, which are well known for mediating recruitment of HSCs (Marra et al., 1999; Schwabe et al., 2003a), SAA has not yet been characterized on its functional role to induce migration of HSCs. In this work, we have identified SAA1 as a *bona fide* chemokine that interacts with TLR2 for mediating migration of HSCs during acute liver inflammatory response. To our best knowledge, this is the first report which firmly identifies that SAA1 engages TLR2 for inducing migration of HSCs.

By using two acute liver injury models, we have demonstrated that expression of SAA1 and TLR2 follows complete colocalization with the recruited HSCs at injury sites. Notably, the *in vivo* depletion of SAA1 in C57/BL6 mice significantly reduced the recruitment of HSCs and altered the expression of TLR2 at the site of injury, indicating that SAA1/TLR2 axis plays a critical role for inducing the recruitment of HSCs during hepatic disease response (Figure 2). However, the chemotactic efficiency of SAA1 appeared to be relatively weaker than PDGF (10 μg/mL vs 10 ng/mL, respectively), a well-known cytokine mediating the migration of HSCs to fibrotic tissues (Yang et al., 2003). This is consistent with previous reports which manifested that chemotactic efficiency of SAA1 varies dose dependently in different cell types, as the maximal chemotactic concentration index was demonstrated to be 300 ng/mL for immature dendritic cells (Gouwy et al., 2015), 8 μM for monocyte and leukocytes (Badolato et al., 1994), and 10 μg/mL for T lymphocytes (Xu et al., 1995).

This work also emphasizes the role of TLR2 as the receptor target for SAA1. The *in vivo* inhibition of TLR2 significantly reduced the migration of HSCs at injury sites, without affecting the expression of SAA1, in both CCl_4_ and CI models (Figure 4). Likewise, the rhSAA1-stimulated LX-2 cells that were lacking TLR2 receptor failed to migrate in the *in vitro* migration assays, which further confirmed that the SAA1/TLR2 axis is critical for promoting HSCs migration toward injury sites.

We extended our study to investigate the underlying molecular mechanism of SAA1/TLR2-mediated migration of HSCs. Recently, it has been described that TLR2 coordinates with the Rho GTPases for multitude cellular activity including cell migration (Manukyan et al., 2009; McGarry et al., 2015). To this context, Rac1 plays a central role in regulating the actin cytoskeleton remodeling during cell migration (Sanz-Moreno and Marshall, 2009). For instance, the activation of Rac1 may induce the MLCK through its phosphorylation at Ser19 which, in turn, acts as the main driver to assemble into bipolar filaments for actomyosin contraction and force generation (Shibata et al., 2015). Our results indicated that SAA1/TLR2 axis induces migration of HSCs through activation of Rac1. The pharmacological inhibition of Rac1 or the gene knockout of TLR2 abolished the SAA1-induced Rac1 activation (Figure 6), which subsequently blocked MLCK phosphorylation, and thus reduced the migration of HSCs. Furthermore, the pharmacological inhibition of PI3K also attenuated SAA1/TLR2-induced migration of LX-2 cells, by blocking the activation of GTPase-Rac1. Indeed, inhibition of PI3K has been reported to block PDGF/PDGFR-induced migration of porcine aortic endothelial cells by attenuating the Rac1 activation (Hooshmand-Rad et al., 1997).

Our results also highlight the contribution of SAA1/TLR2 axis in ECM deposition at injury locus, as we found that SAA1/TLR2 axis promotes collagen deposition by recruiting more activated HSCs at injury locus (Figure 7). In addition, SAA1/TLR2 axis induces inflammation in activated HSCs which is manifested by production of chemokines such as MCP-1, IL-8, and RANTES. Indeed, chemokines are playing critical role in tissue fibrogenesis through inducing their pleiotropic effect by interacting with their cognate receptors, expressed by various target cells (Schwabe et al., 2003a; Wasmuth and Weiskirchen, 2010). For instance, chemokines released by HSCs, in particular RANTES, may exert autocrine and paracrine effects on the nearby HSCs, which subsequently leads to proliferation and recruitment of additional HSCs to the site of injury, a phenomenon that is crucial for hepatic fibrogenesis (Schwabe et al., 2003a). Therefore, SAA1/TLR2 axis-mediated secretion of chemokines from activated HSCs likely contributes for hepatic fibrogenesis and provides a link for cross talk between hepatocytes and HSCs present at the injured liver.

In summary, this work demonstrates that the SAA1/TLR2 axis is a critical mediator for migration of HSCs during acute liver injury. Based on the data described above, one may infer that SAA1 contributes for hepatic fibrogenesis through attracting activated HSCs at injury sites through a signal transduction pathway, in which, Rac1 serves as a downstream effector of PI3K leading to SAA1-mediated actin reorganization and chemotaxis. Finally, excessive recruitment and deposition of HSCs at injury sites is a key driver of liver cirrhosis suggesting that SAA1/TLR2 axis may provide a novel target to anti-fibrosis drug development.

### Limitation of the study

The RNAi approaches we used in this study, although caused significant knockdown of SAA1 probably due to its massive expression pattern in acute liver injury, do not meant to fully eliminate the expression patterns of SAA1 in mice liver. Therefore, we recommend that the future studies should endorse SAA1 knockout mice for further mechanistic studies. However, there is currently no SAA1 knockout mice model available which itself lends a challenge for scientific community.

## STAR★Methods

### Key resources table

**Table:** 

REAGENT or RESOURCE	SOURCE	IDENTIFIER
**Antibodies**		

Anti-α-smooth muscle actin marker	Abcam	Cat# ab21027; RRID:AB_1951138
Anti-α-smooth muscle actin marker	Abcam	Cat#ab5694; RRID:Addgene_80089
Human/mouse/rat α-smooth muscle actin marker	R&D Systems	Cat#MAB1420; Cat#MAB1420
Anti-SAA1/SAA2 antibody [FPR19235]	Abcam	Cat#ab199030; RRID:Addgene_80089
Anti-SAA1/SAA2 antibody [FPR19550]	Abcam	Cat#ab207445; RRID:Addgene_80089
SAA (SAA1): sc-52211	Santa Cruz Biotechnology	Cat#sc-52211; RRID:Addgene_80089
Anti-SAA1 antibody	EpiGentek	Cat#A70907; RRID:Addgene_80089
Anti-TLR2 antibody [T2.5]	Abcam	Cat#ab16894; RRID:Addgene_80089
Anti-TLR2 antibody [EPR20302-119]	Abcam	Cat#ab209216; RRID:Addgene_80089
Toll-like Receptor 2 (D7G9Z) Rabbit mAb #12276	Cell Signaling Technology	Cat#12276; RRID:Addgene_80089
PI3K Kinase p85 (19H8) Rabbit mAb	Cell Signaling Technology	Cat#4257; RRID:Addgene_80089
Anti-PI 3 Kinase p85/p55 (phospho Y199 + Y467) antibody	Abcam	Cat#ab226842; RRID:Addgene_80089
Myosin light-chain 2/MLC-2V rabbit antibody	Proteintech	Cat#55462-1AP; RRID:Addgene_80089
MYLK2 Rabbit antibody	Proteintech	Cat#21173-1-AP; RRID:Addgene_80089
Anti-Myosin light chain (phospho S20) antibody (ab2480)	Abcam	Cat#ab2480; RRID:Addgene_80089
Anti-TLR4 antibody (ab13556)	Abcam	Cat# ab13556; RRID:AB_300457
AGER antibody	Proteintech	Cat#Ag23105; RRID:Addgene_80089
FPR2 antibody	Proteintech	Cat#13448-1-AP; RRID:Addgene_80089
GAPDH mouse monoclonal	Proteintech	Cat#60004-1-Ig; RRID:Addgene_80089
Donkey anti-Rat, Alexa Fluor 594	Thermo Fisher Scientific	Cat #A-21209; RRID:AB_2535795
Donkey anti-Goat, Alexa Fluor 488	Thermo Fisher Scientific	Cat #A-11055; RRID:AB_2534102
Donkey anti-Mouse, Alexa Fluor 568	Thermo Fisher Scientific	Cat # A10037; RRID:AB_2534013
Donkey anti-Mouse, Alexa Fluor 488	Thermo Fisher Scientific	Cat # A-21202; RRID:AB_141607
Goat anti-rabbit	KangChen Bio-tech	Cat #KC-RB-035; RRID:AB_2631281
Goat anti-mouse	KangChen Bio-tech	Cat #KC-MM-035; RRID:AB_2665472
ImmPRESS-AP Horse Anti-Rabbit IgG Polymer Kit	VECTOR	Cat#MP-5401
Anti-Rabbit IgG	Vector Labs	Cat # MP-7401

**Chemicals, peptides, and recombinant proteins**		

Recombinant Human Apo- SAA1	PeproTech	Cat #300-53
Recombinant mouse serum amyloid A1	R&D Systems	Cat #2948-SA-025
PI3K (LY294002)	Selleck.cn	Cat # S1105
NF-ΚB (SC75741)	Selleck.cn	Cat # S7273
Rac GTPase (NSC 23766)	Selleck.cn	Cat #S8031
TLR2 (CU_CPT22)	Selleck.cn	Cat # S8677
RAGE (FPS-ZM1)	R&D Systems	Cat # 6237/10
FPR2 (WRW4)	R&D Systems	Cat # 2262/1
TLR4 (TAK-242)	Selleck.cn	Cat # S7455
RIPA buffer (10 X)	Cell Signaling Technology	Cat# 9806
Protease Inhibitor Cocktail (100 X)	Cell Signaling Technology	Cat# 5871
Phosphatase Inhibitor Cocktail (100 X)	Cell Signaling Technology	Cat# 5870
Isopropanol	DAMAO Co., Ltd.	N/A
Methanol	XIHUA Co., Ltd.	N/A
Ethanol	XIHUA Co., Ltd.	N/A
Acetic acid	Tian in Fuyu Fine Chemical Co., Ltd.	N/A
Chloroform	Tian in Fuyu Fine Chemical Co., Ltd.	N/A
HCl	Tian in Fuyu Fine Chemical Co., Ltd.	N/A
Xylene	Tianjinshi Baishi Chemical Co., Ltd.	N/A
DMEM, low glucose	Thermo Fisher Scientific	Cat# 11885084
DMEM, high glucose	Thermo Fisher Scientific	Cat# 11885084
Penicillin-Streptomycin	Thermo Fisher Scientific	Cat# 15070063
Fetal bovine serum	Thermo Fisher Scientific	Cat# 26140079
D hank	Cienry	N/A
Phosphate buffer saline PBS	Genom	N/A

**Critical commercial assays**		

Human CCL2/MCP-1 Quantikine ELISA Kit	R&D Systems	Cat# DCP00
Human CCL5/RANTES Quantikine ELISA Kit	R&D Systems	Cat#DRN00B
Human IL-8/CXCL8 Quantikine ELISA Kit	R&D Systems	Cat#D8000C
ImmPACT® Vector® Red Substrate Kit	VECTOR	Cat#SK-5105
ImmPACT™ DAB Peroxidase Substrate Kit	VECTOR	Cat#SK-4105
Rho/Rac/Cdc42 Combo Activation Assay kit	Abcam	Cat# ab211168
Pierce™ Classic Magnetic IP/Co-IP Kit	Thermo Fisher Scientific	Cat#88804
PierceTM Silver Stain for Mass Spectrometry	Thermo Fisher Scientific	Cat#24600
Pierce™ BCA Protein Assay Kit	Thermo Fisher Scientific	Cat#23225
ReverTraAce Qpcr ^RT^ Master Mix with gDNA Remover	Toyobo Co., Ltd. Life Science Department, Osaka, Japan	Cat#FSQ-301
KOD-Plus-Neo	Toyobo Co., Ltd. Life Science Department, Osaka, Japan	Cat#KOD-401
Invivofectamine™3.0 Reagent	Thermo Fisher Scientific	Cat#IVF3001
Lipofectamine™ 3000 Transfection Reagent	Thermo Fisher Scientific	Cat# L3000001
TRIzol™ Reagent	Thermo Fisher Scientific	Cat#15596026
Lipofectamine® RNAiMAX Transfection Reagent	Thermo Fisher Scientific	Cat#13778075
TaqMan™ Gene Expression Master Mix	Thermo Fisher Scientific	Cat# 4369016
RNeasy Mini Kit	QIAGEN	Cat# 74106
EndoFree Mini Plasmid Kit II	TIANGEN	Cat#DP118-02
TIANamp Genomic DNA Kit	TIANGEN	Cat#DP304-03
Dual-Luciferase® Reporter Assay System	Promega	Cat#E1910
Picro Sirius Red Stain Kit ab150681	Abcam	Cat#ab150681
Vectamount mounting medium	Vector Labs	Cat##H-5000
Pierce DAB substrate kit	Thermo Fisher Scientific	Cat#34002
ImmPACT Vector Red Substrate Kit	VECTOR	Cat#SK-5105

**Experimental models: Cell lines**		

LX-2/human hepatic stellate cell line	Merck Millipore	Cat#SCC064
Hep-G2/human hepatoblastoma cell line	Merck Millipore	Cat# SCC249
JS1/human immortalized hepatic stellate cell line	Generous gift from Dr. Jin-sheng Guo from Zhongshan Hospital, Fudan University, Shanghai	N/A

**Experimental models: Organisms/strains**		

NOD-SCID-IL2RG^−/−^ mice (NSI mice)	Guangzhou Institute of Biomedicine and Health	N/A
C57BL/6 mice	Vital River Laboratory Animal Technology Co. (Beijing, China)	N/A
Wistar rats	Vital River Laboratory Animal Technology Co. (Beijing, China)	N/A

**Oligonucleotides**		

Stealth siRNA for mouse SAA1 (Table S1)	Life Technologies	N/A
siRNA for rat TLR2 (Table S2)	RiboBio	N/A
Primers (Table S3)	IGEbio	N/A

**Recombinant DNA**		

pGL4.32[luc2P/NF-κB-RE/Hygro]	Promega	Cat#E849A
pGL4.75 [hRluc/CMV] Renilla luciferasecat	Promega	Cat#E2311
41,815, Addgene	Addgene	Cat#41815
pWPXLd-luciferase-EGFP	Addgene	Cat# 12258
pCDH-CMV-MCS-EF1-copGFP-T2A-Puro (SAA1 over expression)	IGEbio	N/A
pCDH-CMV-MCS-EF1-copGFP-T2A-Puro (GFP)	IGEbio	N/A

**Software and algorithms**		

ImageJ	FUJIFILM Corporation	N/A
GraphPad Prism	www.graphpad.com	N/A
Inkscape	Inkscape.org	N/A
SnapGene Viewer	www.snapgene.com	N/A
Motic Images Plus	www.motic.com	N/A

**Other**		

Confocal imaging system	Zeiss 710 NLO	N/A
CFX Connect Real-Time System	Bio-Rad	N/A
Live cell imaging system	Differential phase contrast microscope (Zeiss)	N/A
FACS Aria IIU	BD Biosciences	N/A
NanoDrop ND-1000 N/A	Thermo Fisher Scientific	N/A

### Resource availability

#### Lead contact

Further requests for resources should be directed to the lead contact, Yin-xiong Li (li_yinxiong@gibh.ac.cn)

#### Materials availability

This work did not generate new unique reagents. However, comprehensive protocols for immunostainings are available upon request.

#### Data and code availability

This article includes all analyzed data.

### Experimental model and subject details

#### *In vivo* animal study

##### Mice

All mice used in this study were male and were 6-8 weeks of age. Immune-deficient NOD-SCID-IL2RG^−/−^ mice (NSI mice, GIBH) were obtained from Guangzhou Institute of Biomedicine and Health. Male C57BL/6 mice were purchased from Vital River Laboratory Animal Technology Co. (Beijing, China).

##### Rat

Six to eight weeks male Wistar rats were purchased from Vital River Laboratory Animal Technology Co. (Beijing, China). Mice and rats were maintained on a 12 hour-light/12 hour-dark cycle with free access to food and water. All animal experiments were performed under sterile conditions based upon procedures approved by Guangzhou Provincial Department of Science and Technology (ethical process No: N2014050).

#### Cell lines and primary cell cultures

##### Cell lines

Commercially available human hepatic stellate cell line (LX-2) (merckmillipore, Cat#SCC06) and immortalized mouse hepatic stellate cell line (JS1) (kindly gifted by Dr. Jin-sheng Guo from Zhongshan Hospital, Fudan University, Shanghai) were used for *in vitro* studies. The cells were cultured in high glucose (4.5 g/L) DMEM supplemented with 10% fetal bovine serum (FBS) (Gibco, Thermo Fisher Scientific) and 1% Penicillin-Streptomycin (Thermo Fisher Scientific). Human hepatocytes cell line (HepG2) were cultured in low glucose (1.5 g/L) DMEM supplemented with 10% heat-inactivated fetal bovine serum (FBS) (Gibco, Thermo Fisher Scientific) and 1% Penicillin-Streptomycin. The cultured cells were maintained at 5% CO_2_ in humidified chamber at 37 ⁰C.

##### Primary cells

Primary rat hepatic stellate cells (PRHSCs) and primary mouse hepatocytes (PMHep) were isolated by using a collagenase perfusion and density gradient centrifugation system. Freshly isolated cells were seeded in Dulbecco’s modified Eagle’s medium (DMEM) (Thermo Fisher Scientific) supplemented with 15% fetal bovine serum (FBS) (Gibco, Thermo Fisher Scientific) and 1% Penicillin-Streptomycin (Thermo Fisher Scientific). Hepatic stellate cells isolated from the rats were not passaged and used as culture activated between 7-10 days after isolation.

### Method details

#### Mice and rat

Six to eight weeks male immune-deficient NOD-SCID-IL2RG^−/−^ mice (NSI mice, GIBH) were obtained from Guangzhou Institute of Biomedicine and Health, Chinese Academy of Sciences. Six to eight weeks male C57BL/6 mice and Wistar rats were purchased from Vital River Laboratory Animal Technology Co. (Beijing, China). Mice were maintained on a 12 hour-light/12 hour-dark cycle with free access to food and water. All animal experiments were performed under ethical conditions based upon procedures approved by Guangzhou Providential Department of Sciences and Technology (ethical process No: N2014050).

#### Acute liver injury models

Two models were used to induce acute liver injury in mice. CCl_4_ mediated liver injury was induced by single intraperitoneal (IP) injection of 20% CCl_4_ diluted in olive oil or olive oil alone as vehicle. For cryoinjury model, we used our recently reported method (Abbas et al., 2020). Briefly, metallic cryoprobe was immersed in liquid nitrogen and carefully placed on the left lob of the mice liver until 0.5 cm length of localized lesion was achieved.

#### *In vivo* knockdown of SAA1 using RNAi technology

For *in vivo* knockdown of SAA1, stealth RNAi™ siRNAs were combined with a lipid based delivery reagent invivofectamine3.0 for making complex and, the complexes were injected into C57BL/6 mice at 1.5mg/kg according to the manufacturer instruction (Life technologies). In brief, 250 μl siRNA (3 mg/ml) was mixed with 250 μl complexation buffer. 500 μl of invivofectamine 3.0 reagent was mixed by vortexing to form a complex. The complex was then incubated for 30 min at 50°C. Fourteen milliliter PBS was added to the complex and centrifuged for 1 hr at 3,000 xg (LABOGENE). Finally, the liquid containing the siRNA/invivofectamine complex was adjusted to 0.5 ml with PBS to form a final concentration of 1.4 mg/ml of complexed siRNA. Six to eight week-old mice were given the tail vein injection of siRNA directed against SAA1 or nonspecific control siRNA in to the lateral caudal vein.

#### *In vivo* inhibition of TLR2

For *in vivo* inhibition of TLR2 in mice liver, C57BL/6 mice were intraperitoneally injected CU-CPT22, a TLR2 antagonist (Selleck.cn). In brief, the mice were IP injected 3 mg/kg of CU-CPT22 initially 24 hr before injury and the same dosage was administered again few minutes before inducing CCl_4_ and/or CI injuries.

#### Immunohistochemistry and immunofluorescence

Harvested liver tissues were maintained in 4% paraformaldehyde, embedded in paraffin blocks and sliced 4 μm for immunohistochemistry and 6 μm for immunofluorescence. The slides were deparaffinized in xylene and graded alcohol and antigen retrieval was performed. Antigen retrieval was performed by incubating tissue sections with citrate-EDTA buffer (Beyotime: #P0086) at high fire in microwave oven for about 5 min. Blocking of endogenous peroxidase activity was performed by incubation of the tissues in 0.3% hydrogen peroxide solution for 10 min, followed by incubation in 10% fatal bovine serum (FBS) 1 hr at RT for non-specific binding. All primary antibodies were incubated for 2 hr at RT such as α-smooth muscle actin marker (ab124964, 1:900; ab21027, 1:500, MAB1420, 1:500), TLR2 monoclonal antibody (ab209216; 1:500; ab16894, 1:200) and SAA1 monoclonal antibody (ab199030; 1:1000). The signal was developed by incubating secondary antibodies (Goat anti-Rabbit 488, Goat anti-mouse 488, Goat anti-rabbit 568, Goat anti-mouse 568 (abcam) and anti-Rabbit IgG (Vector Labs # MP-7401)] for 1 hr at RT. The color developed by Pierce DAB substrate kit (# 34002) and counter-stained by DAPI (1:2000) or Hematoxylin solution (sigma) and mounted by Vectamount mounting medium (Vector Labs # H-5000).

#### Sirius red staining

Histological visualization of liver fibrosis induction in mice liver Sirius red staining was performed by (Picro Sirius Red Stain Kit ab150681) according to the manufacturer instruction. In brief, deparaffinized sections of tissue were rehydrated in distilled water for about 5 min. Tissue sections fully covered with picro-sirius red solution and incubated for 60 min. Next, the slides were washed with acetic acid solution absolute alcohol and the slides were mounted with mounting media (Vector Labs # H-5000).

#### Cell culture

For *in vitro* experiments, we used primary rat hepatic stellate cells (PRHSCs), mouse primary hepatocytes (MPHep.), commercially available human hepatic stellate cell line (LX-2) (merckmillipore, Cat#SCC06) and immortalized mouse hepatic stellate cell line (JS1) (kindly gifted by Dr. Jin-sheng Guo from Zhongshan Hospital, Fudan University, Shanghai (Guo et al., 2009; Zhang et al., 2012).

##### LX-2 and JS1 cell culture

LX-2 and JS1 cells were cultured in high glucose Dulbecco’s modified Eagle medium (DMEM)medium supplemented with 5 % FBS (Gibco, Thermo Fisher Scientific) and 1 % Penicillin-Streptomycin (Thermo Fisher Scientific). Cells were cultured in humidified chamber in 5% CO2 at 37 ⁰C

##### Human hepatocytes cell line (HepG2)

HepG2 cells were cultured in low glucose (1.5 g/L) DMEM, supplemented with 10% heat-inactivated fetal bovine serum (FBS) (Gibco, Thermo Fisher Scientific) and 1% Penicillin-Streptomycin.

##### Isolation and culture of PRHSCs

PRHSCs were isolated by two step collagenase perfusion and density gradient centrifugation system as described previously (Mederacke et al., 2015). In brief, rat liver was routinely perfused with a solution containing D-Hank’s (calcium free) and heparin for about 15 min followed by digestion by using 0.5 % collagenase containing D-Hank’s solution supplemented with calcium for 30 min. Liver was homogenized with 0.025 % collagenase and 0.005 % DNAase I and incubated for about 30 min at 37°C. The cells were filtered and centrifuged for 2 min at 50 x g to separate parenchymal cells. After centrifugation for 10 min at 300 x g, the supernatant containing the sinusoidal cell was collected and resuspended in the 28.7 % Nycodenz stock solution. The cells were then centrifuged for 17 min at 1400 x g, the floating HSCs at the top of Nycodenz were collected and resuspended in DMEM (Thermo Fisher Scientific) supplemented with 15 % fetal bovine serum (FBS) (Gibco, Thermo Fisher Scientific). Viable cells were screened by trypan blue exclusion staining and the purity of the cell was determined by expression of Desmin marker by using immunocytochemistry. Hepatic stellate cells isolated from the rats were not passaged and used as culture activated between 7-10 days after isolation.

##### Isolation and culture of primary mouse hepatocytes

Primary mice hepatocytes were isolated based on the method described previously (Charni-Natan and Goldstein, 2020). In brief, rat liver was routinely perfused with D-Hank’s (calcium free) and heparin for about 15 min followed by digestion by using 0.5 % collagenase solution supplemented with calcium for 30 min. Liver was homogenized with 0.025 % collagenase for about 30 min at 37°C. The cells suspension was filtered, centrifuged for 2 min at 50 x g and hepatocytes were isolated from the pellets. Viable hepatocytes were screened by trypan blue exclusion dye as well as by attachment to the collagen coated cell culture plates in DMEM (Thermo Fisher Scientific) supplemented with 15 % FBS (Gibco, Thermo Fisher Scientific) and 1 % Penicillin-Streptomycin (Thermo Fisher Scientific).

For subsequent cell culture experiments, recombinant human SAA1 (PeproTech) (1-15 μg/mL) and recombinant mouse SAA1 (R&D system) (1-15 μg/mL) were diluted with DMEM and exposed to cells for indicated time periods after two washes with PBS. For pharmacological inhibition of PI3K - LY294002 (10 μΜ) (Selleck.cn), NF-κB - NSC 23766 (Selleck.cn), GTPase-Rac1 - SC75741 (50 μΜ) (Selleck.cn), TLR2 - CU-CPT22 (1μM) (Selleck.cn), RAGE - FPS-ZM1 (0.5μM) (R&D system), FPR2 - WRW4 (0.25) (R&D system) and TLR4 - TAK-242 (5nM) (Selleck.cn), were diluted in serum free DMEM and used at indicated concentrations.

#### Intrasplenic cell engraftment

Immune-deficient NOD-SCID-IL2RG^−/−^ male mice (NSI mice, GIBH) were used as a recipient mice for engraftment of cells and to track homing of the cells at injury sites. Before LX-2 transplantation, CCl_4_- and cryoinjury-mediated acute liver injuries were induced and LX-2 (1 × 10^6^) cells were transplanted in spleen immediately. Twenty four hours post-transplantation, the cells were monitored and tracked through fluorescently labeled GFP tag of LX-2 through bioluminescence live imaging system and liver section through confocal microscopy (Zeiss 710 NLO).

#### Transfection of siRNA

Culture activated PRHSCs were pre-prepared for transfection of TLR2 siRNA. TLR2 siRNA (IGE) was preincubated with Lipofectamine 3.0 reagent (Invitrogen) for 15 min according to the instructions of the manufacturer (Life science technology). In brief, the complex formed by mixing of Lipofectamine and siRNA was added to the cells to a final concentration of 10 nM. Negative control cells were transfected with non-targeting siRNA or the transfection reagent alone (mock). After 12 hr, the medium was changed and the cells treated with rmSAA for subsequent experiments. The siRNA sequences used in this study are described in (Table S1).

#### CRISPR/Cas9 gene editing

Human TLR2 gene contains 3 exons spanning 307.9 kb on chromosome 12. The short guide RNA (sgRNA) was designed at downstream of starting code of TLR2 exon 3. To generate TLR2 homozygous knockout LX-2 cells, two guide RNAs targeting exon 3 of TLR2 gene was designed by using online available software (https://www.atum.bio/eCommerce/cas9/input). The two sgRNAs were ligated to the U6-sgRNA cloning vector (41,815, Adgene) to form the TLR2 sgRNAs expression plasmids. The two sgRNAs expressing plasmids were co-transfected to LX-2 cell (8 x 10^5^) using Lipofectamine 3000 (Invitrogen). The cells were treated with puro and resistant clones were selected and expanded into 96-well palates to obtain single cells. Next, the individual single cell colonies were sub-cultured and part of each clone was subjected to PCR-amplification and DNA sequencing. Totally five clones were obtained and among these one was homozygous knockout in which two distinct deletions was existed on both alleles based on sequence analysis. To confirm whether the knockout was a biallelic deleted cell line, PCR product amplified from genomic DNA was cloned into a pTA2 vector, transformed into bacteria, ten representative clones were picked and subjected to sequencing. The analysis showed six of them were found to be 118bp deletion on exon 3 of TLR2, whereas the rest four clones exhibited 113bp deletion. Moreover, protein expression was further confirmed by western blot analysis which was null of TLR2 protein.

#### Luciferase reporter gene assay

LX-2 and culture activated PRHSCs were grown in DMEM medium supplemented with FBS (2%) and penicillin-streptomycin (5%) and incubated in 5% CO_2_ atmosphere. 2 x 10^4^ cells per well were transfected with 2 μg/well of reporter plasmid for nuclear factor pGL4.32[luc2P/NF-κB-RE/Hygro] Vector (Promega cat# E849A) using Lipofectamine 3000 (Invitrogen) according to the manufacturer instructions. In all cases, 0.2 ng/well pRL wild-type *Renilla* luciferase (Rluc) control reporter vector (pGL4.75 [hRluc/CMV] *Renilla* luciferasecat Promega #E2311) was used. After 24 hr of incubation, the cells were serum starved for 12 hr and stimulated by rhSAA (PeproTech) and rmSAA (R&D system) (0.5 – 10 μg/ml) for 6 hr. Then, the reporter gene activity was measured by using Dual Luciferase Assay System (Promega) based on instructions provided by manufacturer.

#### RhoA/Rac1 pull-down activation assay

RhoA/Rac1 pull-down assay was performed using RhoA/Rac1/Cdc42 Activation Assay Combo Kit (ab211168). Briefly, after the cultured cells became approximately 80% confluent, the media was replenished and cells were stimulated with rhSAA1 and rmSAA1 (10 μg/ml) for 24 hr. The cells were then harvested by using lysis buffer supplied with the kit and the lysates were transferred in to Eppendorf tubes. Following centrifuge for 14000 xg for 10 min at 4°C, 20 μl EDTA (0.5 M) was added to each tube. The positive and negative control samples were treated with 10 μl of 100x GTPγS and 100x GDP, respectively, and incubated with slow agitation for 30 min at 30°C. Then, 65 μl of 1M MgCl_2_ was used to stop the reaction. Equal amount of Rhotekin RBD/PAK PBD beads were added to each tube containing the cell lysate and incubated at 4°C for 1 hr. Following incubation, the beads were centrifuged at 14,000 xg for 10 sec and washed twice with the lysis buffer. The Rho-GTP was eluted and run on sodium dodecyl sulfate (SDS) PAGE to determine the extent of GTP bound Rac1 in the sample.

#### Co-immunoprecipitation

Co-IP was performed by using Pierce™ Classic Magnetic IP/Co-IP Kit according to the manufacturer instructions (Thermofisher Scientific). In brief, the protein lysate from rhSAA1 treated LX-2 cells was prepared in co-IP buffer. The cell lysate was incubated for pull-down of target proteins with anti-SAA (SAA1) (sc-52211 Santa Cruz Biotechnology) or TLR2 (sc-166900, mouse monoclonal directed against the amino acids 762–780 at the C-terminus of human TLR2) (Santa Cruz Biotechnology) antibodies overnight at 4ºC. Next, binding of the antigen/antibody complex to protein A/G magnetic beads was performed by incubating for 1 hour at RT. Collection of target protein was performed by elution of the antigen/antibody complex. Finally, the protein concentration of each samples were quantified by Pierce™ BCA Protein Assay Kit (Thermofisher Scientific) according to the manufacturer instructions.

#### Western blot analysis

The cells were extracted by the method as described previously (Siegmund et al., 2006). The amount of protein from each sample was quantified and equal quantity aliquots protein was run on 10-17% SDS gels according to the size of the protein. The gels were transferred on to the PVDF membrane and subjected to blocking with dry milk or bovine serum albumin. The blots were incubated with anti-phospho p65 (S536), anti-phospho (Tyr458)-p85, anti-β-actin (all from Cell Signaling Technology), Matrix Remodeling Sampler Kit (Cell Signaling Technology), anti-Rac1 and -RhoA (ab211168), and Myosin Light Chain, MYLK2 (Proteintech), Anti-TLR4 antibody (ab13556), AGER antibody (Proteintech), FPR2 antibody (Proteintech) for 2 hr at RT. The blots were incubated with secondary antibody (Goat anti-rabbit and Goat anti-mouse HRP conjugated (both from abcam) for 1 hr at RT and the bands were visualized by the enhanced chemiluminescence light method (SAGECREATION, Life Sciences). β-actin or glyceraldehyde phosphate dehydrogenase (GAPDH) (Proteintech) antibodies were used to probe the blot to demonstrate equal loading among the samples.

#### ELISA for chemokines

LX-2 (WT and TLR2^-/-^) cells were treated with rhSAA1 for 24 hr. Levels of human IL-8, MCP-1 and RANTES were determined by sandwich ELISA (R&D Systems) as previously described (Schwabe et al., 2003b).

#### Real-time polymerase chain reaction (RT-PCR)

Total cells were extracted by using Trizol reagents as described by the manufacturer (Invitrogen). 2 μg of RNA was reverse transcribed into single stranded cDNA with a final volume of 20 μl of reaction buffer using a reverse transcription kit (TOYOBO CO., LTD). The mRNA expression(s) of targeted gene(s) was/were quantified by using SYBR Green (Thermo Fisher Scientific) with GAPDH as internal control. Relative expressions of target genes were calculated by using comparative threshold cycle Ct method. All sequences of the primers are described in (Table S2).

#### Agarose spot migration assay

Agarose spot migration assay was performed the method described before (Ahmed et al., 2017). In detail, 100 mg low melting point agarose powder was placed in a 100 ml sterilized beaker and diluted to 20 ml with double distilled water to make a final solution of 0.5% concentration. The solution was heated on a hot plate until complete dissolution and taken off and cool down to 40°C. Next, agarose solution contain rhSAA1 was prepared as follow; different concentrations (1 - 15 μg) of rhSAA1 was prepared with the final volume of 20 μl in H_2_O in different Eppendorf tubes. Each 20 μl of rhSAA1 were individually mixed with 180 μL of 0.5% agarose solution at 40 °C. The ends of 200 μL pipets tips were cut with scissor 2mm to facilitate the agarose solution and formation of uniform spot. By using the cut tips, 10 μl of drops of agarose solution (containing rhSAA1, or control H_2_O) were applied onto 6 well culture plates. Then, the culture plate containing the spot was kept under 4°C refrigerator to allow the spot to set. One mL of (1 × 10^6^ cells/mL) LX-2 suspension cells containing different pharmacological inhibition of PI3K - LY294002 (10 μΜ) (Selleck.cn), NF-κB - NSC 23766 (Selleck.cn), GTPase-Rac1 - SC75741 (50 μΜ) (Selleck.cn), TLR2 - CU-CPT22 (1μM) (Selleck.cn), RAGE - FPS-ZM1 (0.5μM) (R&D system), FPR2 - WRW4 (0.25) (R&D system) and TLR4 - TAK-242 (5nM) (Selleck.cn) was added in the culture plates and then incubated at incubated at 37 °C for 12 hr and the spots were analyzed counting the number of individual migrated cells by using a microscope.

#### Time laps video microscopy

By using agarose spot migration assay experiment, LX-2 cells were cultured in 5% CO_2_ and 37°C humidified atmosphere. Images of the migrating live cells were captured by a differential phase contrast microscope (Zeiss), equipped with a digital camera driven by Image pro plus 6.2 software. (Schindelin et al., 2012). Time laps images were captured by 10 or 20 objective lens.

#### Transwell migration assay

Polycarbonate filters (8.0 μm pore size, Thermo Fisher Scientific) were pre-coated with collagen I (Sigma Aldrich) before use to mimic the *in-vivo* environment. Then, a 100 μl suspension of 24 hr serum starved LX-2 cells (1 x 10^5^) was added in the upper chamber of filter insert. For each control experiment, 500 μl of serum free DMEM was added to the lower chamber. For SAA1 mediated migration assay, 500 μl of serum free DMEM was mixed with increasing concentrations (1-15 μg/ml) of rhSAA1 in the lower chamber of the plate. For co-culture experiment, primary mouse hepatocytes (PMHeps) were isolated from injured and healthy mice liver or SAA1 over expressing Hep G2 cells were placed in the lower chamber of culture plate, JS1 and LX-2 cells were placed in the upper part of culture insert respectively. After 6 hr of incubation, the cells were fixed with 4% paraformaldehyde and stained by crystal violet solution. For migrated cells under the filter sides, representative images were taken at 10x field microscopy and determined by counting.

### Quantification and statistical analysis

Quantification of images from IHC, IF and migration assays was performed by using 10 non-overlapping fields from stained tissue sections and the data were analyzed by ImageJ. Statistical analysis for comparison of two groups was done by *t* test. Comparisons of three or more groups were performed by one-way ANOVA and Tukey’s multiple comparison tests using GraphPad Prism 7.

## Supplemental information

Document S1. Figures S1–S3 and Tables S1–S3

Document S2. Article plus supplemental information
