# Supplementary material for: SAA1/TLR2 axis directs chemotactic migration of hepatic stellate cells responding to injury
Source: iScience. 2021 Apr 29;24(5):102483. doi: 10.1016/j.isci.2021.102483 (PMC8169952; doi:10.1016/j.isci.2021.102483)
Supplement: Document S1. Figures S1–S3 and Tables S1–S3 [file mmc1.pdf]

iScience, Volume *24*

Supplemental information

SAA1/TLR2 axis directs chemotactic migration of hepatic stellate cells responding to injury

Anteneh Getachew, Nasir Abbas, Kai You, Zhen Yang, Muzammal Hussain, Xinping Huang, Ziqi Cheng, Shenglin Tan, Jiawang Tao, Xiaorui Yu, Yan Chen, Fan Yang, Tingcai Pan, Yingying Xu, Guosheng Xu, Yuanqi Zhuang, FeiMa Wu, and Yinxiong Li

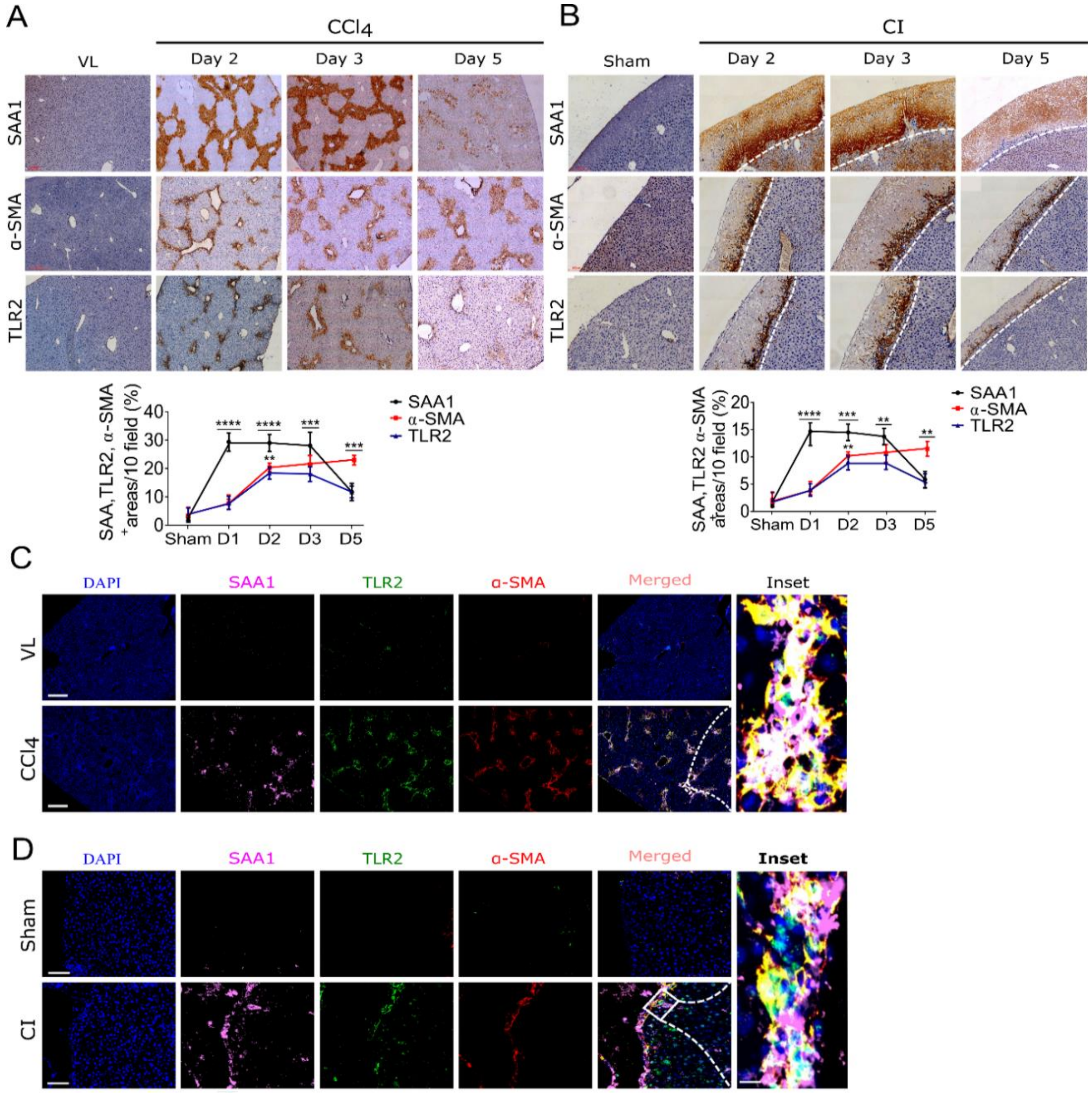

Figure S1. Expression and pathological relevance of SAA1, TLR2 and HSCs at injury locus, Related to Figure 2. (A) Representative IHC staining of SAA1, α-SMA and TLR2 in CCl_4 injury model at indicated time intervals. (B) Representative IHC staining of SAA1, α-SMA and TLR2 in CI injury model at indicated time intervals. Bar graphs represent quantification of IHC positive cells/area in CCl_4 and CI injury model at indicated time intervals. (C and D) Confocal immunofluorescence images showing colocalization of SAA1, TLR2 and HSCs at CCl4 (C) and CI injury (D) models.

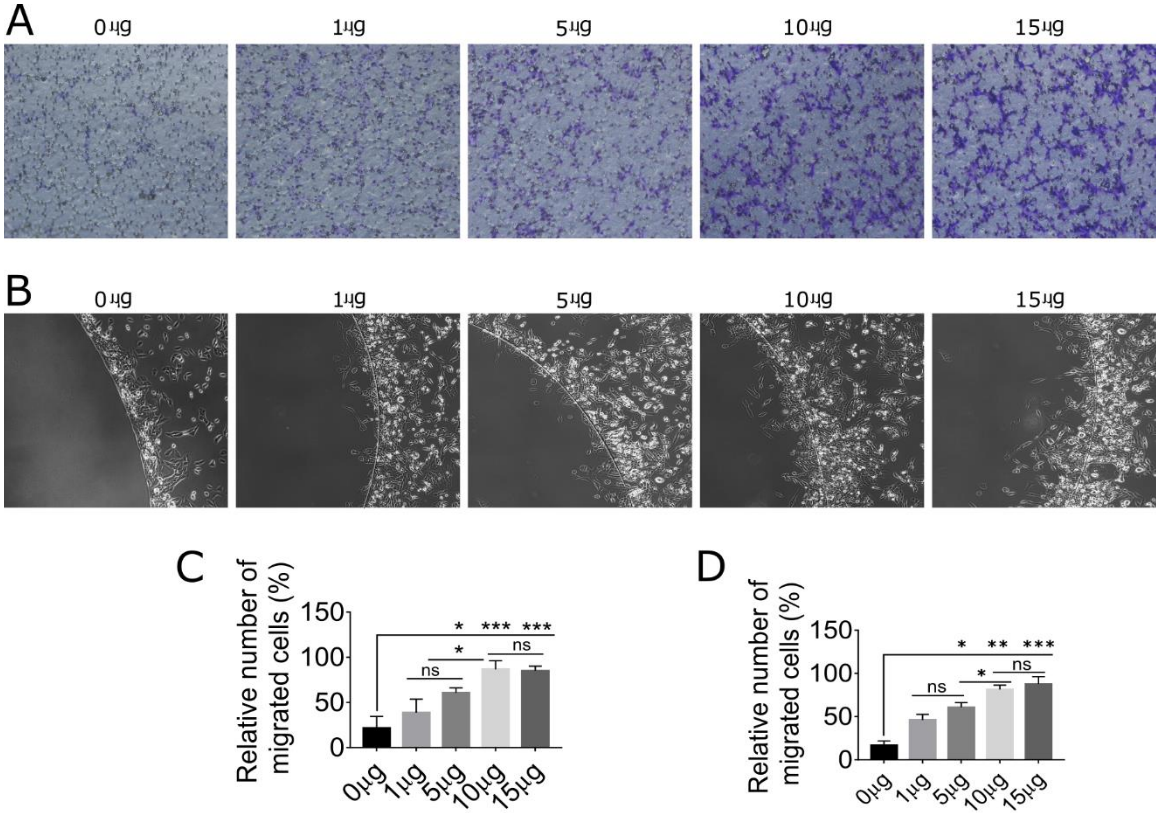

Figure S2. SAA1 mediates migration of HSCs in dose-dependent manner, Related to Figure 2. (A) Representative Transwell migration assays showing SAA1 mediates migration of LX-2 cells in dose dependent (1-15µg) manner. (B) Representative Agdarose spot migration assay showing the cells migrate under SAA1 containing spot in dos-dependent manner at indicated concentrations. (C) Quantification of Transwell migration assay. SAA1 attract LX-2 cells with maximal concentration of 10 to 15 µg/ml. (D) Quantification of agarose migration assay the maximal concentration by which SAA1 attract more numerous cells were shown at 10 to 15µg/ml.

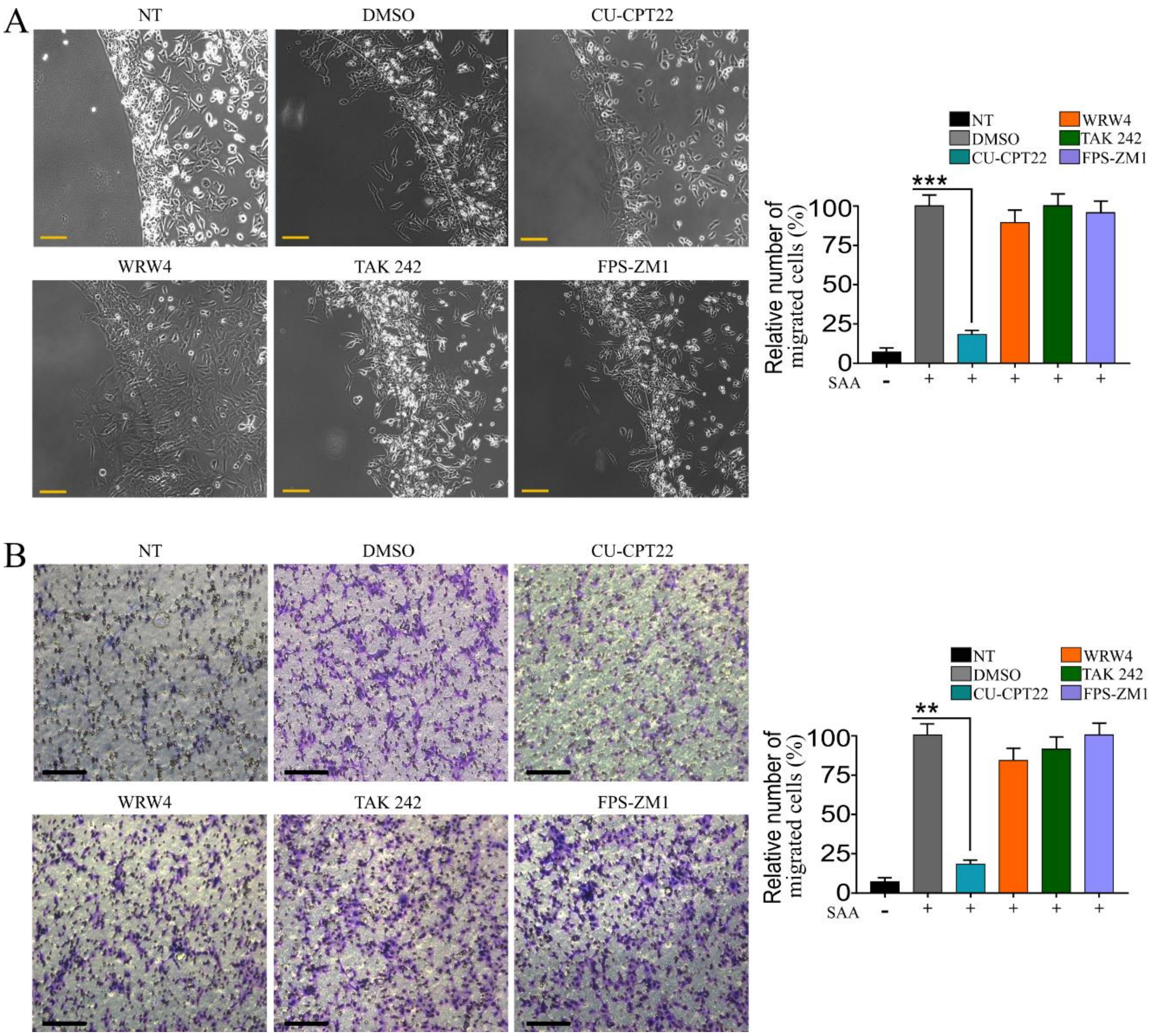

Figure S3. Inhibitor of TLR2 (CU_CPT22) has blocked migration of LX-2 cells in agarose spot and Transwell assays, Related to Figure 3. (A) Agarose spot migration assay showing LX-2 cells pretreated with different inhibitors and the effects in SAA1 induced migration. (B) Transwell migration assay showing LX-2 cells pretreated with different inhibitors and the effects in SAA1 induced migration.

Table S1. Stealth RNAi^{TM} siRNA sequences used for *in vivo* knockdown of SAA1, Related to Figure 1.

SAA1-siRNA1	CAACUAUGAUGCUGCUCAA
SAA1-siRNA2	GUCCUCCUAUUAGCUCAGU
SAA1-siRNA3	GAGGAGAGGGUAAUAAACA
SAA1-siRNA4	CAUAACCUAUUGCUCUCGU
SAA1-siRNA5	GACCUCCUAUUAGCUCACA

Table S2. siRNA sequences used to knockdown of TLR2 in HSCs, Related to STAR methods Transfection of siRNA.

siRNA	（5’-3’）	Modification
Rat-Tlr2-si-1	CAGCAGAAUCAAUACAAUAdTdT	N/A
	UAUUGUAUUGAUUCUGCUGdTdT	
Rat -Tlr2-si-2	GCCUUGACCUGUCUUUCAAdTdT	N/A
	UUGAAAGACAGGUCAAGGCdTdT	
Rat -Tlr2-si-3	GGAAAUGUAGAGACUUUCAdTdT	N/A
	UGAAAGUCUCUACAUUUCCdTdT	
	UAAAUGUUCAAGACUGCCCdTdT	
NC	UUCUCCGAACGUGUCACGUdTdT	N/A
	ACGUGACACGUUCGGAGAAdTdT	
FAM NC	UUCUCCGAACGUGUCACGUdTdT	5’Fam
	ACGUGACACGUUCGGAGAAdTdT	
M/H MAPK1siRNA	UGCUGACUCCAAAGCUCUGdTdT	N/A
	CAGAGCUUUGGAGUCAGCAdTdT	
GAPDH	GUGGAGAUUGUUGCCAUCAdTdT	N/A
	UGAUGGCAACAAUCUCCACdTdT	

Table S3. Primers for used in this study, Related to STAR methods Real-Time Polymerase Chain Reaction.

Primers			Sequences	Gene ID
Species	Gene name			
Human	TLR2	Forward<br>Reverse	gttgcaagcaggatccaaag<br>tgtcttcctgccttcacttgg	ID: 7097
Rat	TLR2	Forward<br>Reverse	aggactcaagagcatcggct<br>gcagaatggccttcccttga	ID: 310553
Human	FPR2	Forward<br>Reverse	agccaagaagcacacaggaa<br>atccgcagaacagtgtagcc	ID: 2358
Rat	FPR2	Forward<br>Reverse	tgctggacgtagcaaacact<br>actcgtaagggacgactgga	ID: 690158
Human	AGER	Forward<br>Reverse	cctcaggaccagggaaccta<br>gaggtctcaccgatgatgct	ID: 177
Rat	AGER	Forward<br>Reverse	agaaaccggtgatgaaggaca<br>ggttgtcgttttcgccacag	ID: 81722
Human	TLR4	Forward<br>Reverse	agacctgtccctgaaccctat<br>cgatggacttctaaaccagcca	ID: 7099
Rat	TLR4	Forward<br>Reverse	gaggacaatgctctggggag<br>atgggttttaggcgcagagt	ID: 29260
Human	SR-B1	Forward<br>Reverse	cctatccccttctatctctccg<br>ggatgttgggcatgacgatgt	ID: 949
Rat	SR-B1	Forward<br>Reverse	gaacacgttctacacgcagc<br>cctgcatggcctccttatcc	ID: 25073
Human	MCP-1	Forward<br>Reverse	cagccagatgcaatcaatgcc<br>tggaatcctgaacccacttct	ID: 6347
Human	IL-8	Forward<br>Reverse	ttttgccaaggagtgctaaaga<br>aaccctctgcacccagttttc	ID: 3576
Human	RANTES	Forward<br>Reverse	ccagcagtcgtctttgtcac<br>ctctgggttggcacacact	ID: 6352
